# Hyaluronic acid-coated Bi:Cu_2_O: an H_2_S-responsive agent for colon cancer with targeted delivery and enhanced photothermal performance

**DOI:** 10.1186/s12951-022-01555-x

**Published:** 2022-07-26

**Authors:** Yuying Cheng, Haiji Bo, Ruomeng Qin, Fulai Chen, Fengfeng Xue, Lu An, Gang Huang, Qiwei Tian

**Affiliations:** 1 Shanghai Key Laboratory of Molecular Imaging, Shanghai University of Medicine and Health Sciences Affiliated Zhoupu Hospital, Shanghai University of Medicine and Health Sciences, Shanghai, 201318 China; 2 Department of Pathology, Naval Medical Center of PLA, No. 338 Huaihai West Road, Shanghai, 200052 China; 3 Shanghai Municipal Education Committee Key Laboratory of Molecular Imaging Probes and Sensors, Shanghai Normal University, Shanghai, 200234 China

**Keywords:** Cu_2_O, Endogenous hydrogen sulfide, CT imaging, Photothermal therapy, Colon cancer

## Abstract

**Background:**

Endogenous hydrogen sulfide (H_2_S)-responsive theranostic agents have attracted extensive attention due to their specificity for colon cancer. However, the development of such agents with high enrichment in tumors and excellent photothermal performance remains challenging.

**Results:**

We prepared hyaluronic acid (HA)-coated Bi-doped cuprous oxide (Bi:Cu_2_O@HA) via a one-pot method. The HA specifically targets colon cancer tumor cells to improve the enrichment of Bi:Cu_2_O@HA at tumor sites, while the doped Bi both enhances the photothermal performance of the H_2_S-triggered Cu_2_O and serves as an agent for tumor imaging. The results in this work demonstrated that the Bi:Cu_2_O@HA nanoparticles exhibit good biocompatibility, target colon cancer tumor cells, facilitate computed tomography imaging, and enhanced H_2_S-responsive photothermal therapy performance, resulting in an excellent therapeutic effect in colon cancer.

**Conclusions:**

The novel Bi:Cu_2_O@HA nanoparticles exhibit excellent tumor targeting and photothermal therapeutic effects, which provide new strategies and insights for colon cancer therapy.

**Graphical Abstract:**

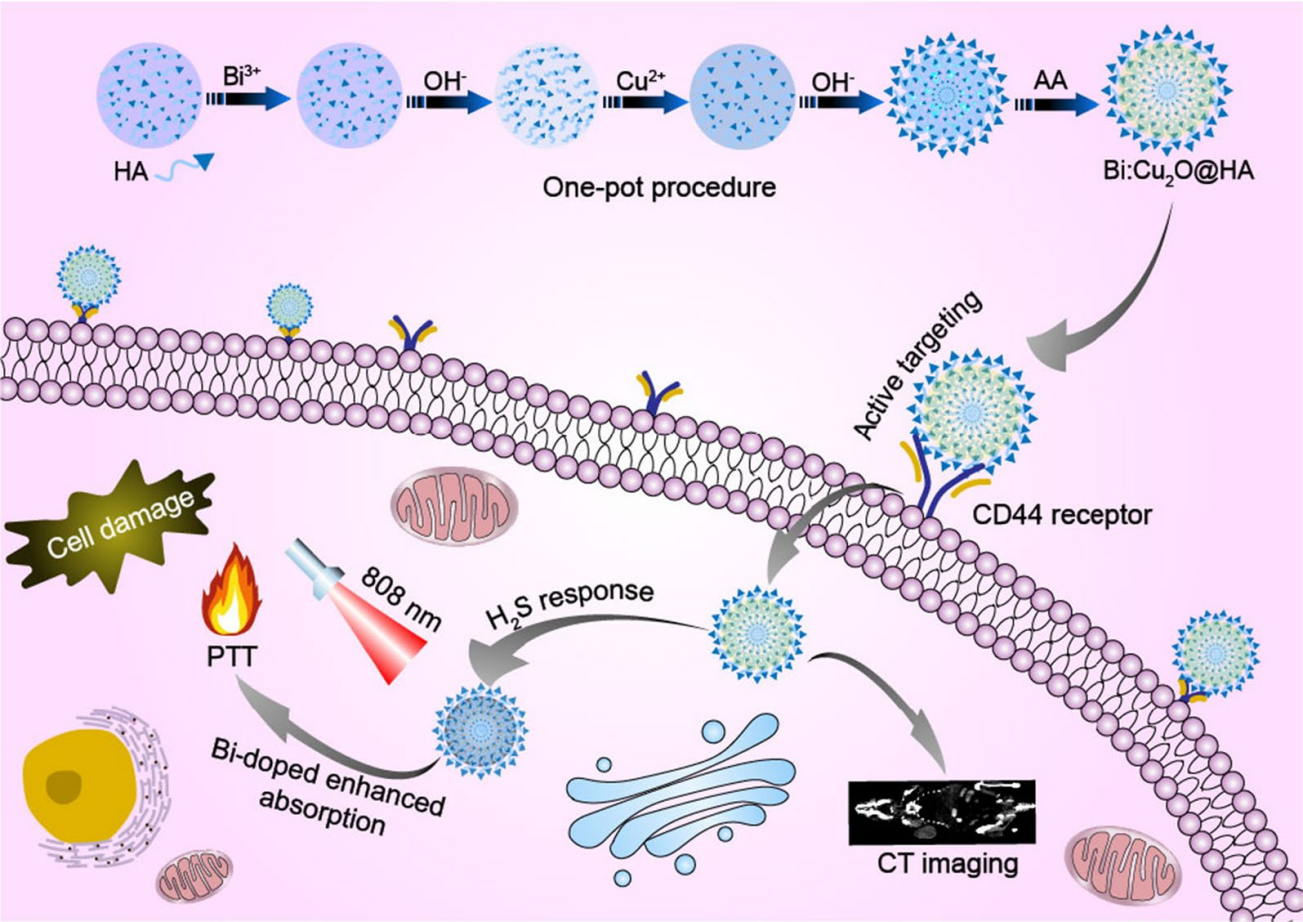

**Supplementary Information:**

The online version contains supplementary material available at 10.1186/s12951-022-01555-x.

## Background

Theranostic agents that are responsive to the tumor environment have attracted considerable attention in diagnosis and treatment due to their ability to target tumor tissues [1–5]. The tumor microenvironment in colon cancer is characterized by the presence of endogenous hydrogen sulfide (H_2_S) [6–9]. Thus, H_2_S can serve as a trigger for colon cancer-targeting theranostic agents. For example, endogenous H_2_S-triggered BODIPY, MOF, and Cu_2_O theranostic agents have been developed for photoacoustic (PA) and near-infrared (NIR) fluorescence imaging along with photothermal and photodynamic therapy for colon cancer [10–12]. However, the enrichment of these agents in tumors depends primarily on the enhanced permeability and retention effect rather than targeted delivery, and their NIR absorption requires further improvement [13, 14]. Therefore, it remains challenging to develop H_2_S-triggered theranostic agents with strong NIR absorption and targeted tumor delivery.

Bismuth (Bi)-based nanoparticles (NPs) are widely used as computed tomography (CT) agents due to their good biocompatibility and X-ray attenuation properties [15–17]. Bismuth sulfide (Bi_2_S_3_) is a narrow-bandgap semiconductor with strong absorption throughout the NIR region [18–21]. Therefore, Bi_2_S_3_ is widely used in combination with other semiconductors to enhance the absorption [22, 23]. For example, the absorption of the Cu_2_O/Bi_2_S_3_ heterojunction is obviously stronger than that of either Cu_2_O or Bi_2_S_3_ alone, especially in the NIR region [24]. Thus, doping with Bi is an effective way to enhance the NIR absorption of endogenous H_2_S-triggered Cu_2_O. However, to the best of our knowledge, little information on this topic is available in the literature.

Hyaluronic acid (HA), a polysaccharide, can target the CD44 receptors that are highly expressed on the surfaces of colon cancer tumor cells [25–28]. Thus, HA is widely used for targeted drug delivery in colon cancer [29–31]. In addition, HA contains abundant carboxyl groups and can act as a surfactant to regulate the synthesis of nanomaterials [32, 33]. Thus, HA can be applied in the one-step synthesis of nanomaterials for targeted tumor delivery. For example, Fu and coworkers developed a one-pot method to prepare MnWO_4_ using HA as a surfactant [34]. The obtained MnWO_4_ NPs exhibited good biocompatibility and tumor-targeting ability. Therefore, HA may be a good surfactant to develop an H_2_S-triggered Bi-doped Cu_2_O theranostic agent with strong NIR absorption and tumor-targeting delivery.

**Scheme 1:**
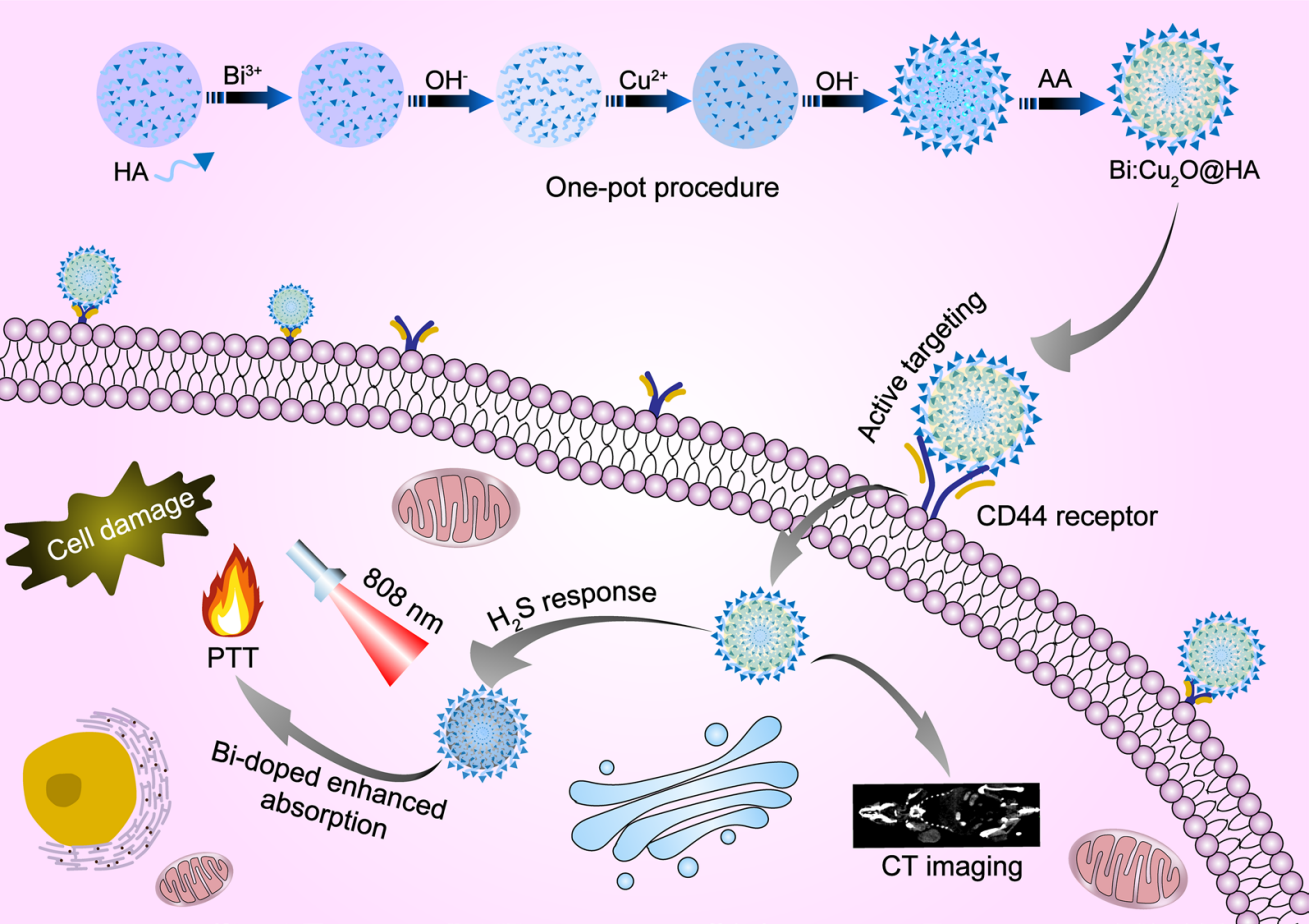
Schematic diagram of the synthesis of multifunctional Bi:Cu_2_O@HA NPs and their application in colon cancer-targeted delivery, CT imaging, and enhanced H_2_S-responsive PTT

As a proof of concept, we constructed a smart H_2_S-responsive nanoplatform: HA-modified Bi-doped cuprous oxide (Cu_2_O) NPs (Bi:Cu_2_O@HA NPs). As shown in Scheme 1, the NPs combine tumor-targeted delivery, high-performance CT imaging, and enhanced photothermal therapy (PTT) into one nanoprobe for the theranostic treatment of colon cancer. The HA not only acts as a surfactant to prepare the NPs, it also improves the tumor-targeting ability of the NPs [35]. Similarly, Bi both improves the CT imaging performance of the NPs and enhances the absorption of Cu_2_O. Thus, the endogenous H_2_S-triggered PTT effect of Cu_2_O can be enhanced by both HA and Bi. This intelligent reagent with tumor-targeted delivery and enhanced theranostic effect opens up a new avenue for developing H_2_S-triggered theranostic agents.

## Results and discussion

### Preparation and characterization of Bi:Cu_2_O@HA NPs

**Fig. 1:**
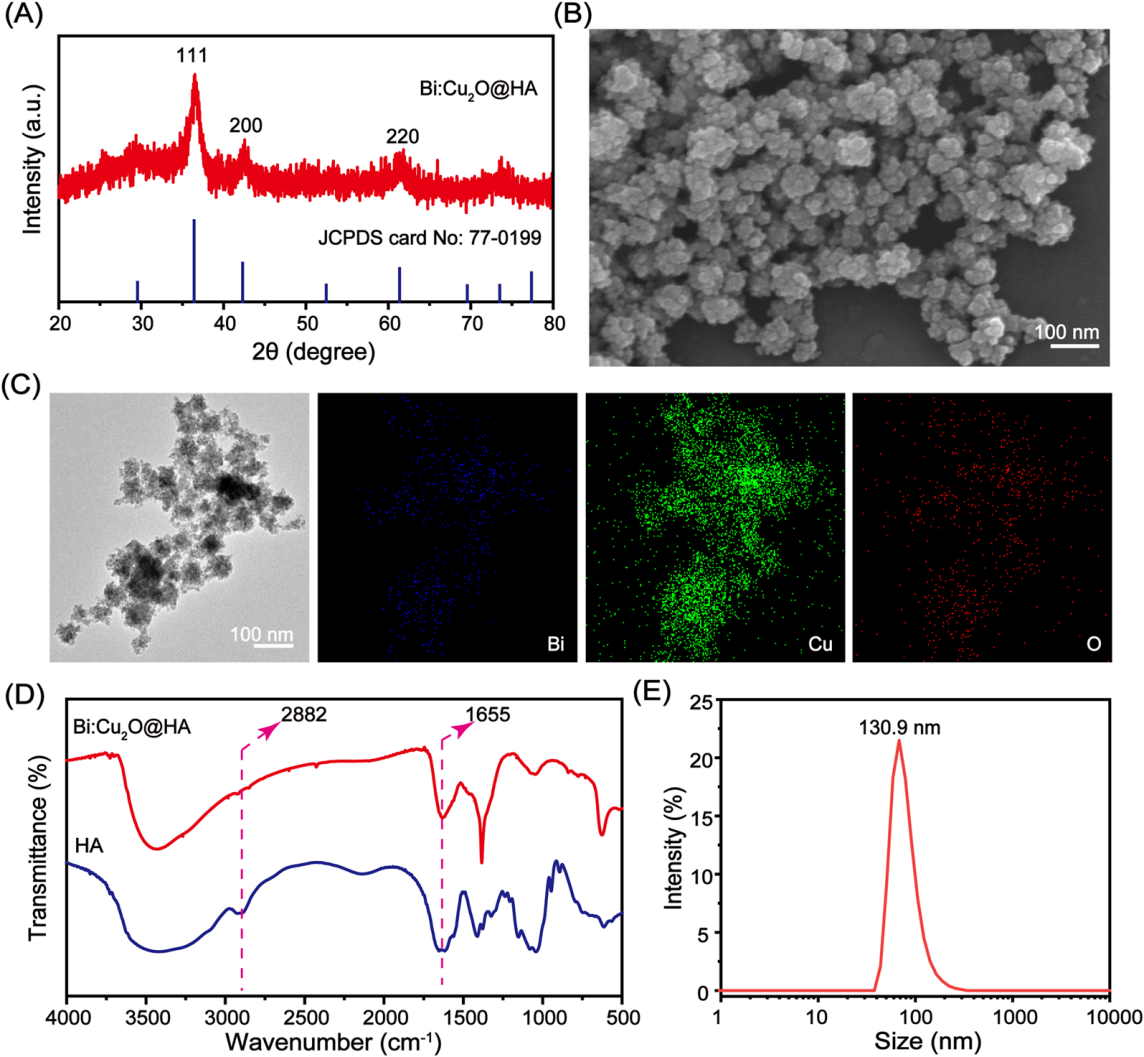
Characterization of Bi:Cu_2_O@HA NPs. **A** XRD pattern (red line). **B** SEM image. **C** TEM image and elemental mapping images. **D** FT-IR spectrum (red line). **E** DLS size distribution of the prepared Bi:Cu_2_O@HA NPs. The blue lines in **A** are the diffraction peaks of cubic Cu_2_O (JCPDS card NO:77–0199). The blue line in **D** is the FT-IR spectrum of HA

The Bi:Cu_2_O@HA NPs were prepared through a one-pot method. After centrifugal purification, the crystalline structure, morphology, composition, and hydrodynamic size of the obtained Bi:Cu_2_O@HA NPs were characterized by X-ray diffraction (XRD), scanning electron microscopy (SEM), transmission electron microscopy (TEM), elemental mapping, Fourier transform infrared (FT-IR) spectroscopy, and dynamic light scattering (DLS). The diffraction peaks of the obtained Bi:Cu_2_O@HA NPs at 36.2, 42.5, and 61.6 degrees are well matched with the (111), (200), and (220) crystal faces of cubic Cu_2_O (JCPDS card NO:77–0199, Fig. 1A), respectively, indicating that the obtained NPs were cubic crystals. As shown in the SEM image (Fig. 1B), the Bi:Cu_2_O@HA NPs had uniform spherical morphologies with particle sizes of approximately 63.09 nm (Additional file 1: Figure S1).

The elemental mapping image (Fig. 1C) demonstrates that Bi, Cu, and O were uniformly distributed in each NP, indicating that Bi was homogeneously doped in the cubic Cu_2_O structure. The X-ray photoelectron spectroscopy (XPS) and energy-dispersive X-ray spectrometry (EDX) results further demonstrated the existence of Bi in the obtained NPs (Additional file 1: Figures S2 and S3). As shown in Fig. 1D, the FT-IR spectrum of HA showed a peak corresponding to the C–H single bond at 2882 cm^− 1^ and a typical amide peak at 1655 cm^− 1^ [36, 37]. These two peaks were retained in the spectrum of Bi:Cu_2_O@HA, indicated that HA was successfully loaded onto the NPs. Furthermore, the zeta potential of Cu_2_O@HA and Bi:Cu_2_O@HA NPs informed the coating of HA on the surface of the obtained NPs (Additional file 1: Figure S4). The hydrodynamic size of the Bi:Cu_2_O@HA NPs determined by DLS was 130.9 nm, much larger than the sizes measured by SEM and TEM (Fig. 1E). This may be due to the strong hydrophilicity of HA. These characterization results demonstrate that hydrophilic Bi:Cu_2_O@HA NPs were successfully prepared. Subsequently, the size and polydispersity index (PDI) changes of Bi:Cu_2_O@HA NPs were studied in water, PBS and serum, respectively (Additional file 1: Figure S5). According to the results, the hydrodynamic diameter did not change significantly within a week, indicating that Bi:Cu_2_O@HA NPs has good dispersion stability.

### H_2_S-responsive performance

**Fig. 2:**
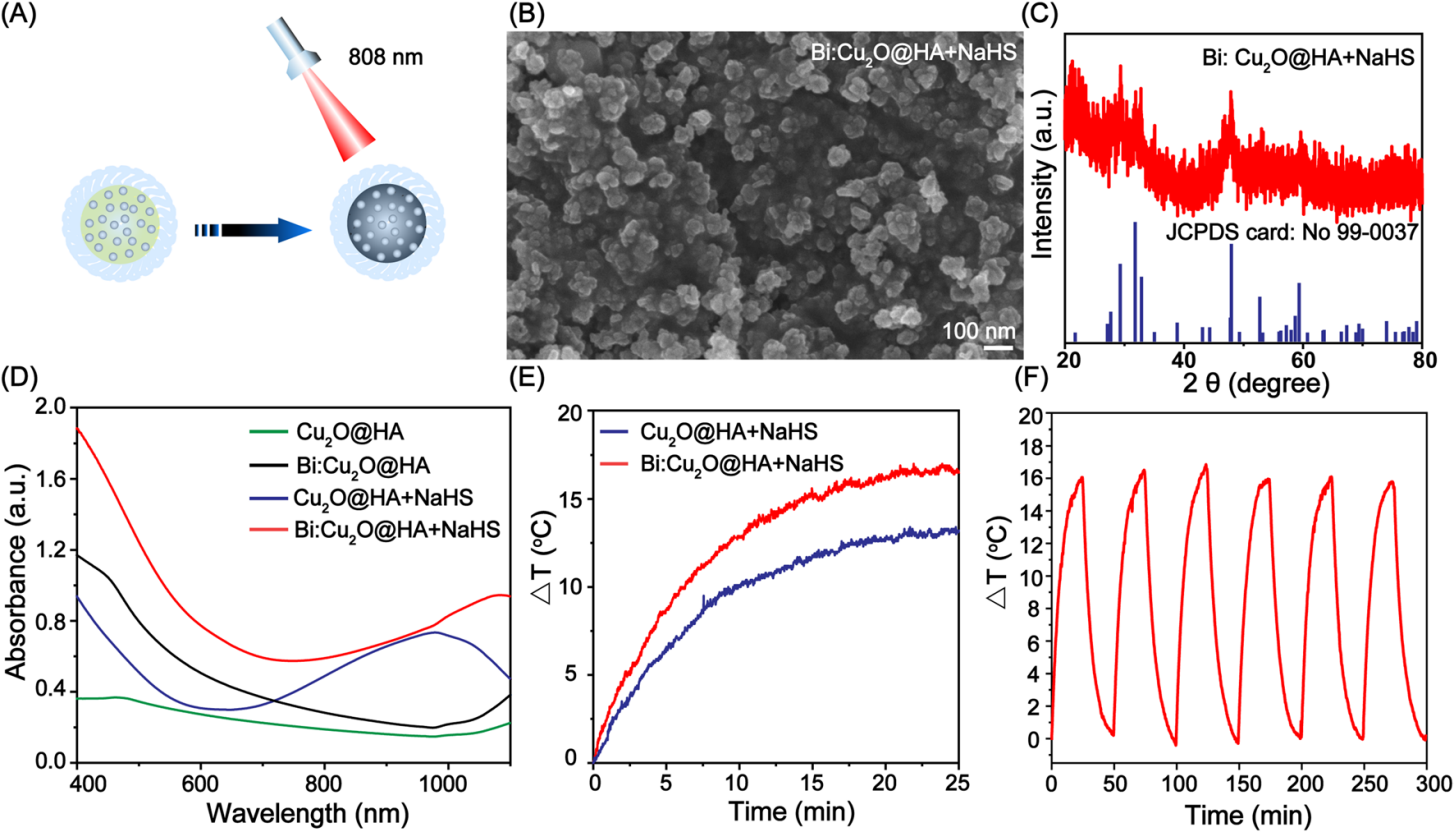
H_2_S-responsive performance of the Bi:Cu_2_O@HA NPs. **A** Schematic diagram of the reaction of Bi:Cu_2_O@HA NPs with NaHS. **B** SEM image. **C **XRD pattern of Bi:Cu_2_O@HA after reaction with NaHS. **D** Absorption of Cu_2_O@HA and Bi:Cu_2_O@HA NPs before and after reaction with NaHS. **E** Plots of Δ*T* vs. time for Cu_2_O@HA + NaHS and Bi:Cu_2_O@HA + NaHS under 808-nm laser irradiation (1 W/cm^2^). **F** Plots of Δ*T* vs. time for Bi:Cu_2_O@HA (0.5 mM) + NaHS (4 mM) during six cycles of irradiation (1 W/cm^2^)

To explore the H_2_S-responsive performance of the Bi:Cu_2_O@HA NPs, NaHS was used to simulate endogenous H_2_S (Fig. 2A), and Cu_2_O@HA NPs prepared using the same method as the Bi:Cu_2_O@HA NPs but without Bi doping were used as a control (Additional file 1: Figures S6–S9). The crystal structure, morphology, absorption, and photothermal performance after reaction with NaHS were investigated. The SEM image in Fig. 2B shows that after reaction with NaHS, the Bi:Cu_2_O@HA NPs exhibited a spherical morphology with an average diameter of approximately 65 nm, slightly larger than the diameter of the initial Bi:Cu_2_O@HA NPs, in agreement with a previous report [38]. In addition, the XRD peaks of the Bi:Cu_2_O@HA NPs after reaction with NaHS at 31.5, 49.5, and 59.4 degrees were well matched with the (103), (110), and (116) crystal faces of hexagonal CuS (JCPDS card NO: 99 − 0037, Fig. 2C), respectively, indicating the formation of CuS.

To investigate whether doping with Bi enhanced the NIR absorption of Cu_2_O, we measured the absorption of the Cu_2_O@HA and Bi:Cu_2_O@HA NPs before and after reaction with NaHS. As shown in Fig. 2D, both the Cu_2_O@HA and Bi:Cu_2_O@HA NPs exhibited stronger absorption in the NIR region after reaction with NaHS compared with before reaction. Notably, the NIR absorption, especially at the laser wavelength of 808 nm (Additional file 1: Figure S10A, B), of Bi-doped Cu_2_O@HA after reaction with NaHS was slightly improved compared with that of Cu_2_O@HA, indicating that doping with Bi has the potential to enhance the photothermal performance of Cu_2_O exposed to H_2_S. The photothermal performances of the Cu_2_O@HA and Bi:Cu_2_O@HA after reaction with NaHS were compared based on the temperature changes (Δ*T*) of dispersions of the NPs in water under irradiation by an 808-nm laser. As shown in Fig. 2E, the Δ*T* values of Cu_2_O@HA and Bi:Cu_2_O@HA respectively increased by 13.2 and 16.5 °C after reaction with NaHS, suggesting that the photothermal performance of Cu_2_O@HA was improved by doping with Bi. The Δ*T* of Bi:Cu_2_O@HA explored by different dispersion concentration and laser power density further suggests the good performance (Additional file 1: Figure S11A–D). The photothermal conversion efficiency also increased slightly compared to the previously reported efficiency for Cu_2_O (Additional file 1: Figure S12A, B) [12]. Furthermore, after six irradiation and cooling cycles (Fig. 2F), the maximum Δ*T* of the Bi:Cu_2_O@HA dispersion after reaction with NaHS hardly changed, indicating the good photothermal stability of Bi:CuS@HA. The above results indicate that Bi doping is an effective strategy to enhance the photothermal performance of H_2_S-responsive Cu_2_O@HA NPs.

### CT imaging and tumor-targeting performance

**Fig. 3:**
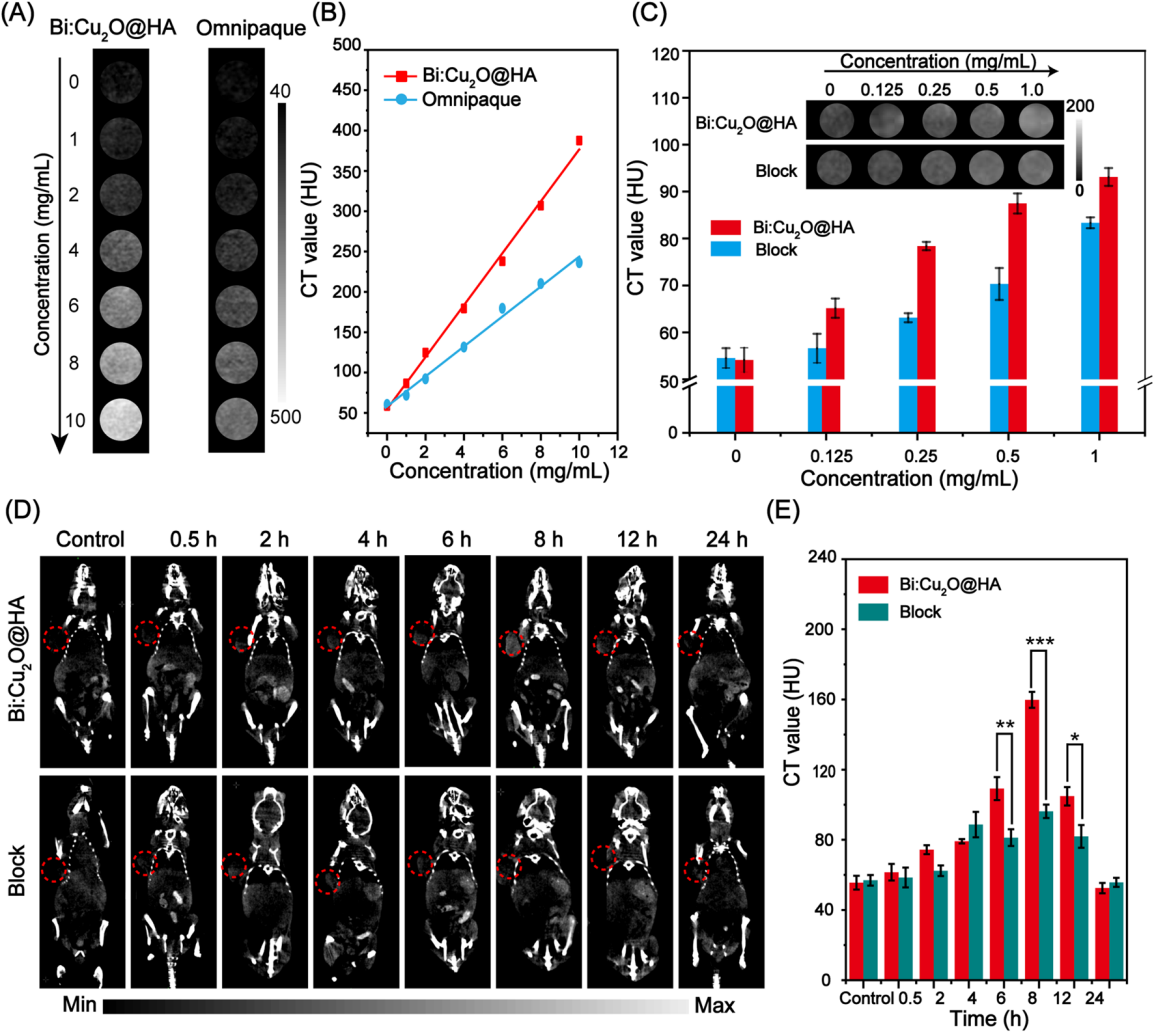
**A**, **B** In vitro CT images and corresponding linear correlations of the CT signals with the concentrations of Bi:Cu_2_O@HA NP and Iohexol. **C** In vitro CT images (inset) and corresponding plots of the CT signals vs. concentration for CT26 cells in the Bi:Cu_2_O@HA and block groups. **D**, **E** In vivo CT images and corresponding CT values at the tumor sites of tumor-bearing mice after injection in the Bi:Cu_2_O@HA and block groups. Data are presented as means ± SDs (n = 3). *p < 0.1, **p < 0.01, ***p < 0.001

Considering the good X-ray attenuation properties, the CT imaging performance of the Bi:Cu_2_O@HA NPs was investigated using the commercial Iohexol CT contrast agent as a control. As shown in Fig. 3A, as the concentrations of Iohexol and Bi:Cu_2_O@HA increased, the CT images of both agents became brighter, indicating a gradual increase in the CT signals. Furthermore, the CT imaging performance of the Bi:Cu_2_O@HA NPs was superior to that of Iohexol at the same concentration. The linear correlations between the CT signals and the concentrations of Iohexol and Bi:Cu_2_O@HA further demonstrate that the CT imaging performance of Bi:Cu_2_O@HA NPs was better than that of Iohexol at the same concentration (Fig. 3B). The above results suggest that Bi:Cu_2_O@HA NPs can be used as an agent for CT imaging.

Based on the good CT imaging performance and the ability of HA to target the highly expressed CT44 receptors on the surfaces of colon cancer cells, the targeting ability of the Bi:Cu_2_O@HA NPs was explored both in vitro and in vivo using CT imaging. Two groups of experiments were established: one with the Bi:Cu_2_O@HA group and another with a block group. As shown in Fig. 3C, the CT image of the CT26 colon cancer cells after incubation with Bi:Cu_2_O@HA was brighter than that of the block group at the same concentration (inset of Fig. 3C). The corresponding signal of the Bi:Cu_2_O@HA group was also much stronger than that of the block group, suggesting that HA significantly enhanced the tumor cell targeting ability. The CT images of tumor-bearing mice were collected after the intravenous injection of Bi:Cu_2_O@HA to evaluate the tumor-targeting performance in vivo.

As shown in Fig. 3D, the colors of the CT images at the tumor sites (red circles) before injection were similar in the Bi:Cu_2_O@HA and block groups. After intravenous administration, the tumor sites in the CT images of the mice in the Bi:Cu_2_O@HA group gradually became brighter and reached maximum brightness at 8 h after injection. In comparison, the tumor sites in the CT images of mice in the block group were darker at the same time points. The corresponding signals at the tumor sites were much higher in the Bi:Cu_2_O@HA group than in the block group (Fig. 3E). These results further indicate that the Bi:Cu_2_O@HA NPs exhibited good targeting performance for colon cancer in vivo since HA can target the expressed receptors on cancer.

### Biocompatibility

**Fig. 4:**
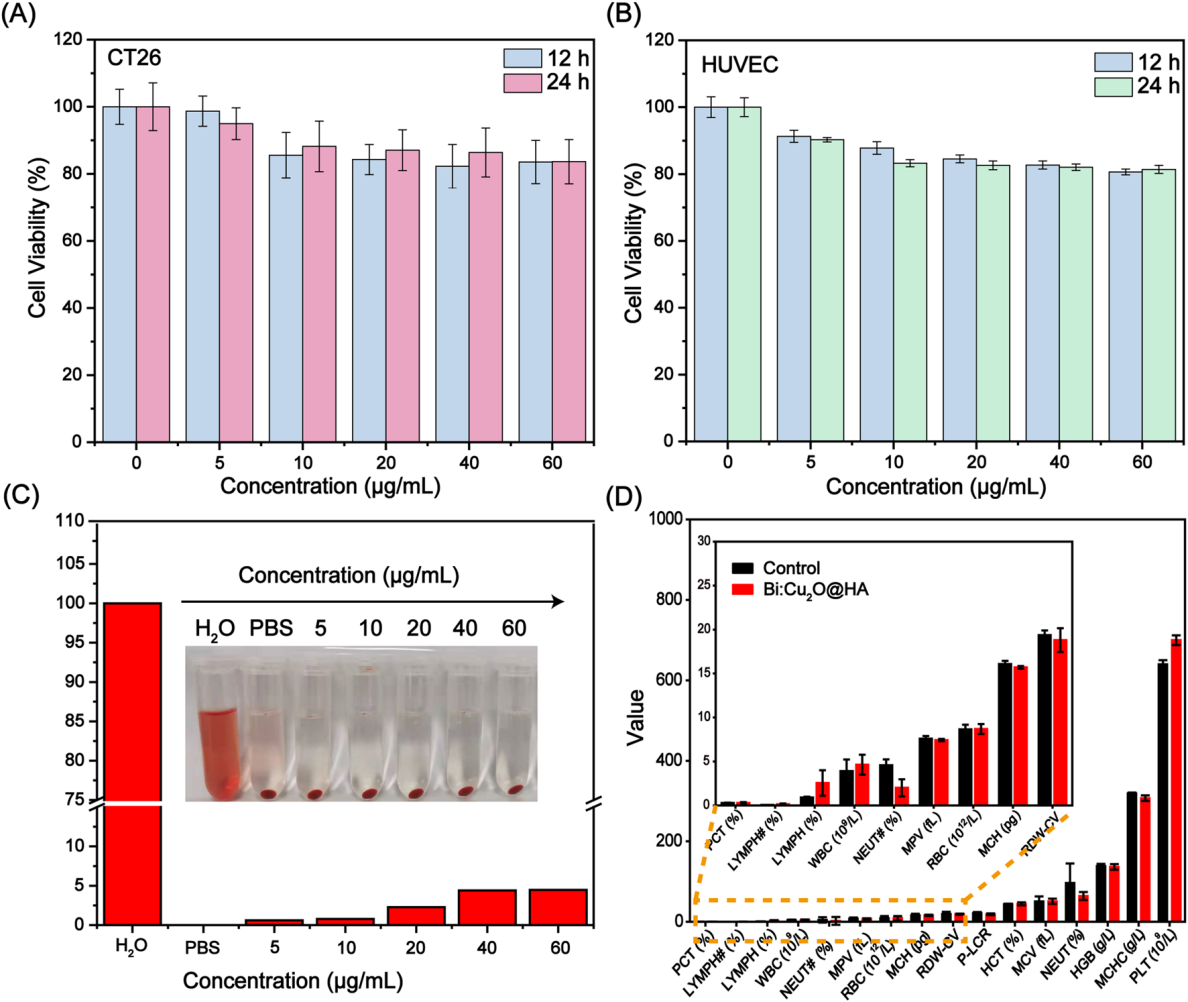
Biocompatibility of Bi:Cu_2_O@HA NPs. **A**, **B** Viability of CT26 cells and HUVEC cells incubated with different concentrations of Bi:Cu_2_O@HA for 12 and 24 h, respectively. **C** Hemolytic effect of Bi:Cu_2_O@HA NPs. **D** Hematological assay data of mice before (Control) and after (Bi:Cu_2_O@HA) intravenous administration of Bi:Cu_2_O@HA NPs

Cytotoxicity, hemolysis, and routine blood biochemical index analyses were performed to investigate the biocompatibility of the Bi:Cu_2_O@HA NPs. First, the cytotoxicity of the Bi:Cu_2_O@HA NPs was assessed in human umbilical vein endothelial cells (HUVECs) and mouse colon cancer CT26 cells by MTT assay. The cell survival rates of both the HUVEC and CT26 cells were more than 80%, even at a concentration of 80 µg/mL (Fig. 4A, B), indicating that the Bi:Cu_2_O@HA NPs had low cytotoxicity. Compared to water (positive control), the Bi:Cu_2_O@HA NPs did not cause significant damage to the erythrocyte membranes (Fig. 4C), similar to the PBS group (negative control). More importantly, the routine blood indexes of the mice after the tail vein injection of Bi:Cu_2_O@HA NPs for 36 h were not significantly different than those of mice in the control group, indicating the good biocompatibility of Bi:Cu_2_O@HA NPs in vivo (Fig. 4D). These results demonstrate that the Bi:Cu_2_O@HA NPs exhibited good biocompatibility and great potential for further application in vivo.

### In vitro PTT

**Fig. 5:**
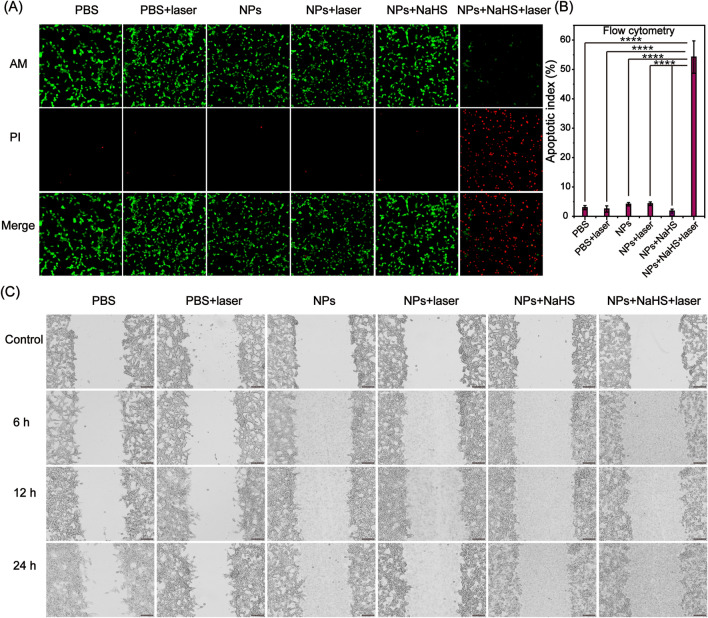
In vitro PTT effect. **A** Confocal laser scanning microscopy images of CT26 cells stained by Calcein AM (green color) and PI (red color). **B** Apoptotic indexes for different groups. Data are presented as means ± SDs (n = 3). ****p < 0.0001. **C** Photographs of the scratched areas after treatment in different groups

To explore the photothermal effect of Bi:Cu_2_O@HA NPs after triggering by H_2_S, the CT26 cells were stained with Calcein-AM (AM) and propidium iodide (PI) to visualize the therapeutic effect, while the apoptosis rate of the cells was evaluated by flow cytometry. The cells in the PBS, NPs, and NPs + NaHS groups were incubated with PBS, NPs, and NPs + NaHS media, respectively, while the cells in the PBS + laser, NPs + laser, and NPs + NaHS + laser groups were additionally subjected to laser irradiation. First, the CT26 cells were stained with Calcein AM (green, live cells) and propidium iodide (PI; red, dead cells), as illustrated in Fig. 5A. In the PBS and NPs groups along with the PBS + laser and NPs + laser groups, red fluorescence was negligible, indicating that these treatments did not cause cell death. Although CuS was generated in the NPs + NaHS group, only a few cells were observed with red fluorescence, indicated that the NPs + NaHS treatment could not induce cell death without laser irradiation. In contrast, most cells in the NPs + NaHS + laser group showed red fluorescence, indicating the good photothermal treatment effect of the H_2_S-activated Bi:Cu_2_O@HA NPs under 808-nm laser irradiation. The apoptosis of CT26 cells in different groups was quantified by flow cytometry (Additional file 1: Figure S13). The apoptosis rates of cells in the PBS, PBS + laser, NPs, NPs + laser, NPs + NaHS, and NPs + NaHS + laser groups were 2.96%, 2.50%, 4.12%, 4.30%, 1.70%, and 54.21%, respectively (Fig. 5B), further indicating that the H_2_S-activated Bi:Cu_2_O@HA NPs effectively induced apoptosis in cancer cells under 808-nm laser irradiation. Next, we studied the in vitro cytotoxic effect of different groups using MTT assay and drawn the same conclusion (Additional file 1: Figure S14). To investigate the photothermal effect of H_2_S-activated Bi:Cu_2_O@HA NPs under 808-nm laser irradiation on cell migration, wound-healing assays were carried out using CT26 colon cancer cells. After scratching, the cells in the PBS, NPs, and NPs + NaHS groups were incubating with PBS, NPs, and NPs + NaHS media for different times, while the PBS + laser, NPs + laser, and NPs + NaHS + laser groups additionally received 5 min of irradiation with an 808-nm laser.

As shown in Fig. 5C, the CT26 cells in the PBS, NPs, NPs + NaHS, PBS + laser, NPs + laser, and NPs + NaHS groups still showed movement in the scratched area, suggesting that these treatments did not significantly affect the migratory ability of CT26 cells. Compared to the other groups, the cells in the NPs + NaHS + laser group barely moved toward the scratched area, indicating that PTT based on the H_2_S-activated Bi:Cu_2_O@HA NPs under 808-nm laser irradiation will obviously inhibit the migration of CT26 cells (Additional file 1: Figure S15). The above results indicate that PTT based on Bi:Cu_2_O@HA NPs triggered by H_2_S can both promote cell apoptosis and inhibit cell migration. Thus, the Bi:Cu_2_O@HA NPs show promise as a nano-agent for the treatment of colon cancer.

### In vivo PTT

**Fig. 6:**
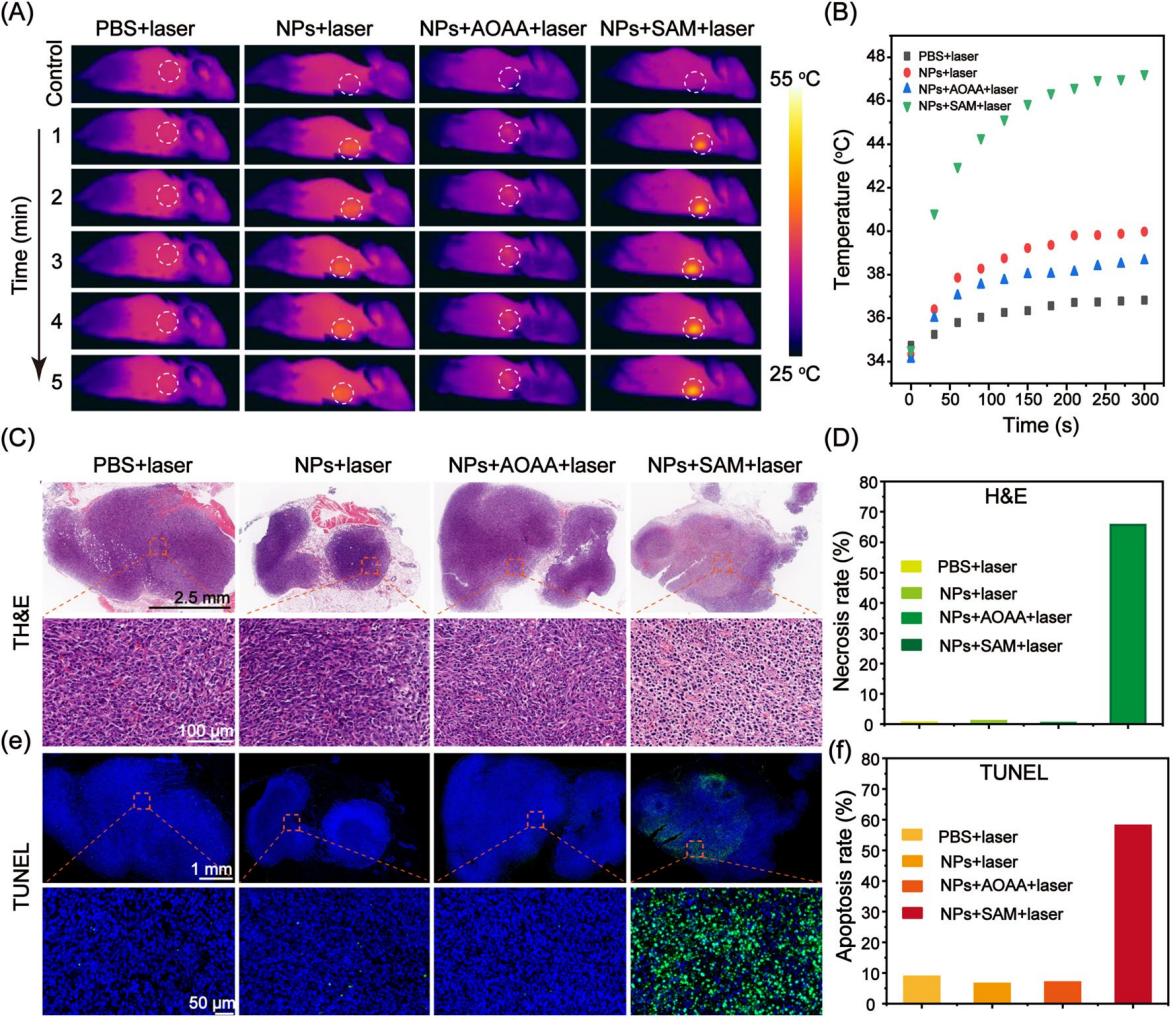
In vivo PTT. **A**, **B** Thermal images and corresponding temperature changes of the mice in different groups under laser irradiation. **C**, **D** Photographs of H&E-stained global and local tumor slices and the corresponding necrosis rates for different groups. **E**, **F** Fluorescence images of TUNEL-stained global and local tumor slices and the corresponding apoptosis rates for different groups

To confirm the tumor ablation effect of the Bi:Cu_2_O@HA NPs in vivo, experiments were carried out in CT26 tumor-bearing mice. First, the mice in the PBS + laser and NPs + laser groups were intravenously injected with PBS and Bi:Cu_2_O@HA NPs, respectively, while the mice in the NPs + AOAA + laser and NPs + SAM + laser groups were also pretreated with AOAA (aminooxyacetic acid, an endogenous H_2_S inhibitor) and SAM (*S*-adenosyl-L-methionine, an endogenous H_2_S promoter), respectively, before injection with Bi:Cu_2_O@HA NPs. According to the CT imaging results, the Bi:Cu_2_O@HA NPs reached the maximum enrichment level in the tumor at 6 h after injection. Therefore, PTT was performed at 6 h after injection, and the temperature changes in the tumor region were monitored using a thermal camera. As shown in Fig. 6A and B, the color of the tumor sites in the PBS + laser, NPs + laser, and NPs + AOAA + laser groups did not change obviously after 5 min of laser irradiation, and the temperature increased from 34.75 to 36.8 °C, 39.98 °C, and 38.65 °C, respectively. In contrast, an obvious color change was observed in the NPs + SAM + laser group, and the temperature increased to 47.23 °C. The large difference between the NPs + SAM + laser group and the other groups demonstrates that the photothermal activity of Bi:Cu_2_O@HA was only activated by the endogenous H_2_S in the colon cancer tumor. After laser treatment, a tumor tissue was randomly dissected from each group, and the necrosis and apoptosis in the tumor tissue were evaluated by H&E and TUNEL staining. H&E staining (Fig. 6C) showed that the tumor tissues in the PBS + laser, NPs + laser, and NPs + AOAA + laser groups were not obviously damaged under laser irradiation. In contrast, a large amount of cell necrosis was observed in the tumors in the NPs + SAM + laser group, and the corresponding positive cell rate was 65.67% (Fig. 6D). According to the TUNEL staining images (Fig. 6E), the tumor slices from the PBS + laser, NPs + laser, and NPs + AOAA + laser groups showed almost no green fluorescence (dead cells), indicating that only a small number of cells were apoptotic In contrast, a large area of green fluorescence was observed in the NPs + SAM + laser group, suggesting that the photothermal effect of the activated Bi:Cu_2_O@HA NPs killed cells in vivo. The corresponding cell apoptosis rates in the PBS + laser, NPs + laser and NPs + AOAA + laser, and NPs + SAM + laser groups were 8.86%, 6.54%, 6.98%, and 58.09%, respectively (Fig. 6F), in agreement with the H&E staining results (Fig. 6D). The above results demonstrate that the photothermal activity of the Bi:Cu_2_O@HA NPs can be triggered by the overexpressed H_2_S in colon cancer cells, and that the NPs exhibit an excellent photothermal therapeutic effect, suggesting that the NPs are a promising candidate for colon cancer therapy.

**Fig. 7:**
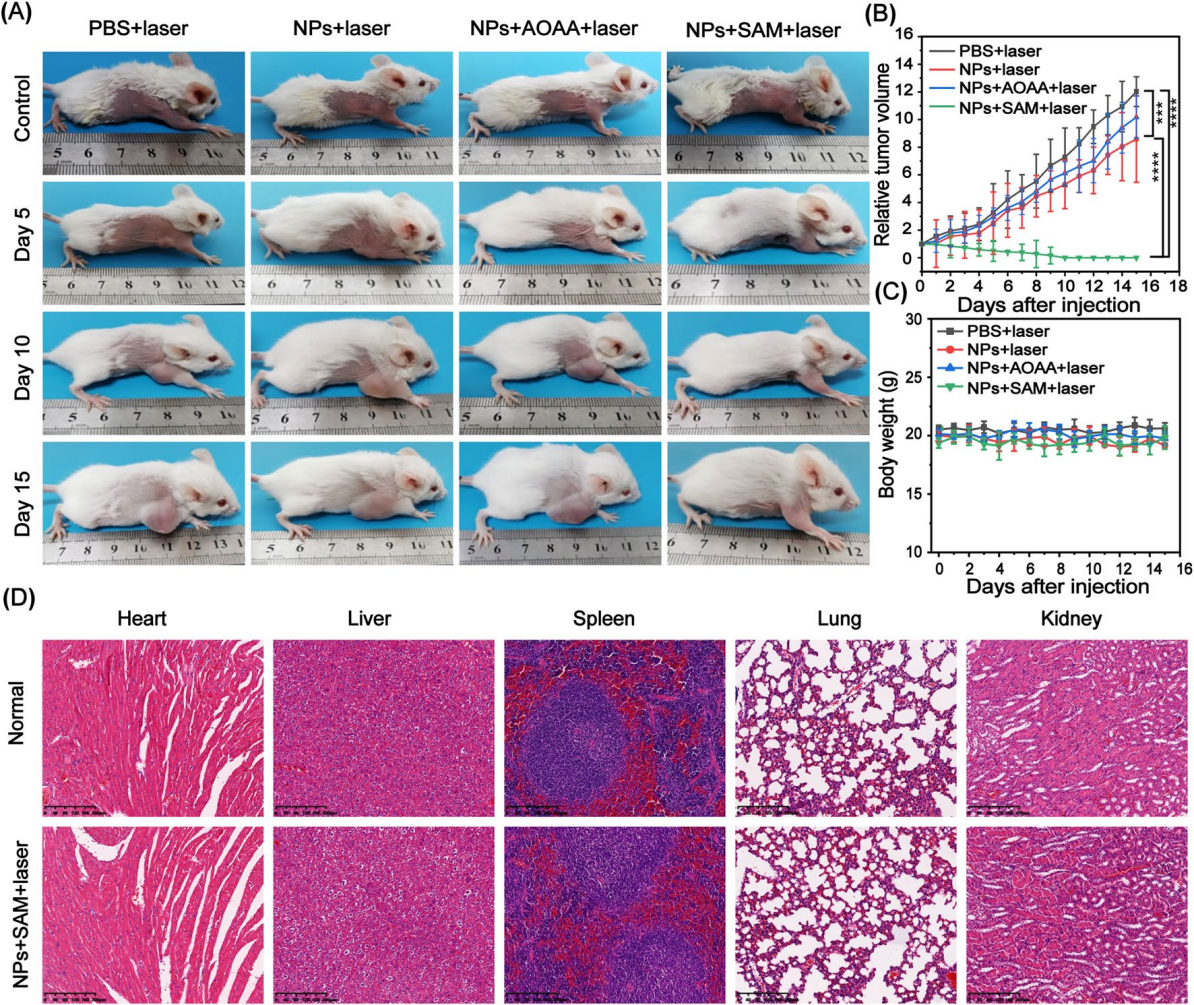
PTT effect in vivo. **A** Images of CT26 tumor-bearing mice collected after 15 days of treatment. **B**, **C** Relative tumor volume and body weights in mice during 15 days of treatment in different groups. Data are presented as means ± SDs (n = 4). ***p < 0.001, ****p < 0.0001. **D** H&E staining images of major organs from normal and cured mice

To evaluate the therapeutic effect of Bi:Cu_2_O@HA NPs in vivo, the state of subsistence and tumor volume of the mice were monitored for 15 d. As demonstrated in Fig. 7A, the tumors of the mice in the PBS + laser, NPs + laser, and NPs + AOAA + laser groups continued to grow rapidly, while the tumors of the mice completely disappeared after 15 d of treatment (Additional file 1: Figure S16). The corresponding changes in the relative tumor volumes for each group revealed similar results (Fig. 7B), indicating that only the activated, photothermally active Bi:Cu_2_O@HA NPs could eliminate the tumors. To evaluate the long-term biocompatibility of the Bi:Cu_2_O@HA NPs in vivo, the body weights of the mice in all groups were monitored for 15 d. Subsequently, one of the cured mice in the NPs + SAM + laser group was euthanized, and its main organs were dissected for comparison with those of normal mice to further evaluate the long-term biocompatibility of Bi:Cu_2_O@HA NPs in vivo. As shown in Fig. 7C, the body weight of the mice did not change significantly during the treatment process, indicating that the Bi:Cu_2_O@HA NPs did not affect the normal life activities of the mice or have obvious toxic or side effects. Notably, in contrast to the normal mice, the H&E-stained sections of the cured mouse showed no obvious signs of tissue necrosis (Fig. 7D), indicating that the Bi:Cu_2_O@HA NPs have good long-term biocompatibility in vivo. These results suggest that the Bi:Cu_2_O@HA NPs show excellent potential for the PTT of colon cancer.

## Conclusions

In summary, Bi:Cu_2_O@HA NPs, an H_2_S-responsive agent with tumor-targeted delivery and an excellent theranostic effect for colon cancer were successfully prepared via a one-pot method. Doping with Bi not only enhances the NIR absorption of the endogenous H_2_S-triggered Cu_2_O, it also serves as a CT agent for tumor imaging. Moreover, HA can specifically target the CD44 receptors that are overexpressed in colon cancer cells, thereby improving the enrichment of the Bi:Cu_2_O NPs in tumors and enhancing the therapeutic effect. The results demonstrate that the H_2_S-activated Bi:Cu_2_O@HA NPs have a good PTT effect without toxic side effects in healthy tissues during the treatment process. The novel Bi:Cu_2_O@HA NPs with enhanced H_2_S-responsive photothermal performance provide a new strategy for targeted colon cancer therapy.

## Materials and methods

### Chemicals and materials

Bismuth nitrate and hyaluronic acid were purchased from Shanghai McLean Biochemical Technology Co., Ltd. Copper acetate and sodium hydroxide were purchased from Sinopharm Chemical Reagent Co., Ltd. (Shanghai, China). Ascorbic acid was purchased from Sigma-Aldrich (St. Louis, MO, USA). Sodium hydrosulfide purchased from Adamas-beta. All chemicals were used directly without further processing.

### Preparation of Bi:Cu_2_O@HA NPs

First, 70 mg HA and 1.0 mL Bi(NO_3_)_3_·5H_2_O (0.14 M) were mixed in 50 mL of deionized water under stirring. Next, 1.0 mL NaOH (2 M) was added slowly to the above mixture, and 1.4 mL Cu(CH_3_COO)_2_ (0.2 M) and 1.0 mL NaOH (2 M) were successively added. Finally, 2.0 mL ascorbic acid (0.1 M) was added, and the mixture was stirred for 10 min. The formed Bi:Cu_2_O@HA NPs were centrifuged to remove excess solvent and washed three times with deionized water.

### Characterization

The morphology of the Bi:Cu_2_O@HA NPs was determined by SEM (Zeiss EVO MA 26/LS 26) and TEM (JEOL JEM-2100). The crystalline structure of the Bi:Cu_2_O@HA NPs was confirmed by XRD (Rigaku, Tokyo, Japan). The UV-VIS-NIR absorbance spectrum was measured using a spectrophotometer (DU 730: Beckman Coulter, Brea, CA, USA). CT imaging was performed using a PET/CT in vivo molecular and preclinical imager (Mediso, Nanoscan PET/CT 82s, Hungary).

### H_2_S-responsive performance of the Bi:Cu_2_O@HA NPs

NaHS was used to simulate endogenous H_2_S. After incubation with NaHS (4 mM) at 37 °C for 90 min, the Bi:Cu_2_O@HA NPs (0.5 mM) were centrifuged for SEM and XRD characterization to explore the changes after H_2_S triggering. The absorption of both the Bi:Cu_2_O@HA NPs (40 µg/mL) and Cu_2_O@HA (40 µg/mL) before and after incubation with NaHS (4 mM) at 37 °C for 90 min was evaluated to determine whether doping with Bi enhanced the absorption of endogenous H_2_S-responsive Cu_2_O in the NIR region. Subsequently, a thermal imager (FLIR A300) was used to measure the temperature changes of the Bi:Cu_2_O@HA NPs (40 µg/mL) and Cu_2_O@HA (40 µg/mL) after vulcanization under 808-nm laser irradiation at 1.0 W/cm^2^ for 25 min. To demonstrate the remarkable photothermal stability of the H_2_S-activated Bi:Cu_2_O@HA NPs, we exposed the vulcanized liquid to 808-nm (1.0 W/cm^2^) laser irradiation for 25 min and then turned off the laser for 25 min; this heating and cooling process was repeated six times. Throughout the heating and cooling cycles, the temperature of the solution was recorded by a thermal camera (FLIR A300).

### CT imaging performance of the Bi:Cu_2_O@HA NPs in vitro

To investigate the CT imaging performance of the Bi:Cu_2_O@HA NPs, the same concentrations (1, 2, 4, 6, 8, and 10 mg/mL) of the prepared Bi:Cu_2_O@HA NPs and Iohexol were placed in 2-mL EP tubes for in vitro CT imaging. PBS was used as a control (0 mg/mL).

### Targeting ability of the Bi:Cu_2_O@HA NPs in vitro

The targeting experiments were divided into two groups: the Bi:Cu_2_O@HA group was treated with 2 mL of Bi:Cu_2_O@HA NPs (0.125, 0.25, 0.5, and 1.0 mg/mL); and the block group was treated with a mixture of 1 mL each of HA and Bi:Cu_2_O@HA NPs (0.25, 0.5, 1.0, 2.0 mg/mL). The two groups were incubated with CT26 cells for 12 h followed by the collection of CT images, respectively. Three wells were set up for each concentration, and DMEM high-glucose medium was used as a control (0 mg/mL).

### Biocompatibility of the Bi:Cu_2_O@HA NPs

Cytotoxicity assay: The CT26 cells were first incubated with DMEM (control) or a Bi:Cu_2_O@HA DMEM dispersion with a concentration of 5, 10, 20, 40, or 60 µg/mL for 12 or 24 h. Next, 10 µL of MTT was added and cultured in a cell incubator for 4 h. Subsequently, 100 µL of formazan solution was added to the well and cultured in the cell incubator for another 4 h. Finally, the absorbance at 570 nm was measured using a microplate reader. Five parallel groups were set up for each concentration.

Hemolysis test: A suspension of red blood cells in PBS (0.4 mL, 2%) was prepared following a previous report [39] and added into 1 mL of PBS buffer solution (negative group), deionized water (positive group), or a Bi:Cu_2_O@HA NPs PBS dispersion with a concentration of 5, 10, 20, 40, or 60 µg/mL. After 4 h, the mixture was centrifuged, and the absorbance of the supernatant in each group was measured at 541 nm. The hemolysis rate was calculated following a previous report [40, 41]. Three parallel samples were established in each group.

Routine blood analysis: The mice were randomly divided into two groups with three mice per group. One group was intravenously injected with PBS (100 µL), while the other group was injected with 100 µL Bi:Cu_2_O@HA (10 mg/kg). After 36 h, the blood was collected for routine blood analysis.

### In vitro PTT with Bi:Cu_2_O@HA NPs

The grouping and treatment of cells were the same for all three assays to investigate the photothermal effect of the Bi:Cu_2_O@HA NPs. Namely, the groups were the PBS, PBS + laser, NPs, NPs + laser, NPs + NaHS, and NPs + NaHS + laser groups. The cells in the PBS, NPs, and NPs + NaHS group were incubated with DMEM, Bi:Cu_2_O@HA NPs, and Bi:Cu_2_O@HA NPs + NaHS, respectively. The laser groups additionally received irradiation with an 808-nm laser at 1 W/cm^2^ for 5 min. The concentrations of Bi:Cu_2_O@HA and NaHS in the NPs, NPs + laser, NPs + NaHS, and NPs + NaHS + laser groups were both 0.4 mM.

Calcein-AM/PI double-staining assay: The cells were incubated in six-well plates. After 6 h of treatment, the medium solution in the well was removed and washed with PBS. The cells were then stained by Calcein AM-PI work solution (5 mL of PBS with 10 µL of Calcein-AM and 15 µL of PI). After 15 min, the AM-PI work solution in the well was removed and washed by DMEM for observation by confocal laser scanning microscopy.

Flow cytometry assay: The cells were incubated in six-well plates. After 6 h of treatment, the cells were collected in a centrifuge tube by digestion for staining using the staining solution (100 µL 1 × buffer with 5 µL FITC and 5 µL PI). After staining, the cells were transferred into a flow tube with a strainer for flow cytometry using Novocyte 2000 (Agilent).

Wound-healing assay: The cells were incubated in six-well plates. Before treatment, one clear line was scratched, and a photograph was taken in each group for use as a control. After 6 h of treatment, the culture medium was removed and washed with PBS to eliminate floating cells. The scratched line was recorded by taking a photograph using the same scale as for the control. The medium was then replaced with fresh medium for the next 6 h of incubation. The above steps were repeated to collect photographs of the scratched line after 6, 12, and 24 h of incubation in each group.

In vitro toxicity assay: Cells were cultured overnight in 96-well plates. After 6 h of treatment in different groups, 10 µL of MTT solution was added to each well to continue culturing cells for 4 h. After that, dimethyl sulfoxide (100 µL) was added and shaken gently to dissolve the crystals. Measure the absorbance of the solution at 570 nm on a microplate reader.

### Tumor models

Female mice aged 5–6 weeks old were purchased from Shanghai SLAC Laboratory Animal Co., Ltd. The CT26 tumor-bearing mouse model was established by subcutaneously injecting 1 × 10^6^ CT26 tumor cell suspension into the right upper limb of each mouse. When the tumor volume reached 100 mm^3^, follow-up experiments were started in the mice.

### In vivo targeting and CT imaging performance of Bi:Cu_2_O@HA

The CT26 tumor-bearing mice were randomly divided into two groups: the Bi:Cu_2_O@HA group was intravenously injected with Bi:Cu_2_O@HA NPs (10 mg/kg), while the block group was first intravenously injected with HA (50 µL, 1 mg/mL) followed by the intravenous injection of Bi:Cu_2_O@HA NPs (10 mg/kg) half an hour later. The CT images of the mice were collected before injection (control) and at 0.5, 2, 4, 6, 8, 12, and 24 h after injection.

### In vivo photothermal imaging and PTT

The CT26 tumor-bearing mice were randomly divided into four groups (PBS + laser, NPs + laser, NPs + AOAA + laser, and NPs + SAM + laser) with five mice in each group. For the PBS + laser and NPs + laser groups, the mice were injected with PBS (100 µL) and Bi:Cu_2_O@HA NPs (10 mg/kg), respectively. The mice in the NPs + AOAA + laser and NPs + SAM + laser groups were first injected with AOAA (100 µL, 0.5 mg/mL) and SAM (100 µL, 5 mg/mL) in the abdominal cavity and then injected with Bi:Cu_2_O@HA NPs (10 mg/kg) after 12 h. At 6 h after each injection, the tumor was irradiated with an 808-nm laser (1 W/cm^2^) for 5 min. A thermal imaging camera was used to record the photothermal images in the different groups. Finally, one mouse was randomly selected from each group, and the tumor tissue was dissected for H&E and TUNEL staining after the photothermal treatment. For the remaining groups of mice (*n* = 4), the body weights and tumor volumes were recorded over 15 d.

## Supplementary Information

**Additional file 1: Figure S1**. Size statistics of the Bi:Cu_2_O@HA NPs in a SEM image. **Figure S2.** XPS spectrum of the Bi:Cu_2_O@HA NPs. **Figure S3.** EDX analysis spectrum of the Bi:Cu_2_O@HA NPs. **Figure S4.** Zeta potentials of Cu_2_O@HA NPs and Bi:Cu_2_O@HA NPs. **Figure S5.** (A) Physiological stability of Bi:Cu_2_O@HA NPs in water. (B) Physiological stability of Bi:Cu_2_O@HA NPs in PBS. (C) Physiological stability of Bi:Cu_2_O@HA NPs in serum. (D) Corrsponding PDI change within 7 days. **Figure S6.** SEM of the Cu_2_O@HA NPs. **Figure S7.** DLS size distributions of the Cu_2_O@HA NPs. **Figure S8.** XRD pattern of Cu_2_O@HA (red line). **Figure S9.** FT-IR spectra of HA (black line) and Cu_2_O@HA NPs (red line). **Figure S10.** (A) UV-vis spectra of Bi:Cu_2_O@HA NPs reacted with NaHS for different time periods. (B) Corresponding absorption values at 808 nm. **Figure S11.** (A) Thermal images of the reaction of 4 mM NaHS with different concentrations of Bi:Cu_2_O@HA NPs (808 nm, 1 W/cm^2^). (B) Thermal images of the reaction of 4 mM NaHA with 0.5 mM Bi:Cu_2_O@HA NPs under different power density. (C) Plots of Δ*T* vs. time for different concentrations Bi:Cu_2_O@HA NPs reacted with 4 mM NaHS (808 nm, 1 W/cm^2^). (D) Plots of Δ*T* vs. time for 0.5 mM Bi:Cu_2_O@HA NPs reacted with 4 mM NaHS under different laser power densities. **Figure S12.** (A) Heating and cooling curves of water and Bi:Cu_2_O@HA + NaHS (4 mM) with the laser on and off. (B) corresponding time constant of the cooling curve. (The photothermal conversion efficiency of Bi:Cu_2_O@HA after reacted with NaHS was calculated to be 16.96%.) **Figure S13.** Apoptosis in CT26 cells after treatment in different groups. **Figure S14.** The cell viability of CT26 after treatment in different groups. Data are presented as means ± SDs (n = 5). ****p < 0.0001. **Figure S15.** Cell migration rate of CT26 cells after treatment in different groups for 6 h, 12 and 24 h. **Figure S16.** Photos of the tumors from mice in different groups after treatment.

## Data Availability

All data generated or analyzed during this study are included in this article.
