# Supplementary material for: Hyaluronic acid-coated Bi:Cu2O: an H2S-responsive agent for colon cancer with targeted delivery and enhanced photothermal performance
Source: J Nanobiotechnology. 2022 Jul 26;20:346. doi: 10.1186/s12951-022-01555-x (PMC9327345; doi:10.1186/s12951-022-01555-x)
Supplement: Supplementary file 1 — Additional file 1: Figure S1. Size statistics of the Bi:Cu2O@HA NPs in a SEM image. Figure S2. XPS spectrum of the Bi:Cu2O@HA NPs. Figure S3. EDX analysis spectrum of the Bi:Cu2O@HA NPs. Figure S4. Zeta potentials of Cu2O@HA NPs and Bi:Cu2O@HA NPs. Figure S5. (A) Physiological stability of Bi:Cu2O@HA NPs in water. (B) Physiological stability of Bi:Cu2O@HA NPs in PBS. (C) Physiological stability of Bi:Cu2O@HA NPs in serum. (D) Corrsponding PDI change within 7 days. Figure S6. SEM of the Cu2O@HA NPs. Figure S7. DLS size distributions of the Cu2O@HA NPs. Figure S8. XRD pattern of Cu2O@HA (red line). Figure S9. FT-IR spectra of HA (black line) and Cu2O@HA NPs (red line). Figure S10. (A) UV-vis spectra of Bi:Cu2O@HA NPs reacted with NaHS for different time periods. (B) Corresponding absorption values at 808 nm. Figure S11. (A) Thermal images of the reaction of 4 mM NaHS with different concentrations of Bi:Cu2O@HA NPs (808 nm, 1 W/cm2). (B) Thermal images of the reaction of 4 mM NaHA with 0.5 mM Bi:Cu2O@HA NPs under different power density. (C) Plots of ΔT vs. time for different concentrations Bi:Cu2O@HA NPs reacted with 4 mM NaHS (808 nm, 1 W/cm2). (D) Plots of ΔT vs. time for 0.5 mM Bi:Cu2O@HA NPs reacted with 4 mM NaHS under different laser power densities. Figure S12. (A) Heating and cooling curves of water and Bi:Cu2O@HA + NaHS (4 mM) with the laser on and off. (B) corresponding time constant of the cooling curve. (The photothermal conversion efficiency of Bi:Cu2O@HA after reacted with NaHS was calculated to be 16.96%.) Figure S13. Apoptosis in CT26 cells after treatment in different groups. Figure S14. The cell viability of CT26 after treatment in different groups. Data are presented as means ± SDs (n = 5). ****p < 0.0001. Figure S15. Cell migration rate of CT26 cells after treatment in different groups for 6 h, 12 and 24 h. Figure S16. Photos of the tumors from mice in different groups after treatment. [file 12951_2022_1555_MOESM1_ESM.docx]

Supporting Information

Hyaluronic acid-Coated Bi:Cu_2_O: an H_2_S-Responsive Agent for Colon Cancer with Targeted Delivery and Enhanced Photothermal Performance

Yuying Cheng†^,1，3^, Haiji Bo†^,2^, Ruomeng Qin^1^, Fulai Chen^1^, Fengfeng Xue*^,1^, Lu An^3^, Gang Huang*^,1^, Qiwei Tian*^,1^

† These authors contributed equally to this work.

*E-mail: Xueff@sumhs.edu.cn; [huangg@sumhs.edu.cn](mailto:huangg@sumhs.edu.cn); tianqw@sumhs.edu.cn

^1^Shanghai Key Laboratory of Molecular Imaging, Jiading District Central Hospital Affiliated Shanghai University of Medicine and Health Sciences, Shanghai University of Medicine and Health Sciences, Shanghai 201318, China.

^2^Department of Pathology, Naval Medical Center of PLA, No. 338 Huaihai West Road, Shanghai, 200052, China.

^3^Shanghai Municipal Education Committee Key Laboratory of Molecular Imaging Probes and Sensors, Shanghai Normal University, Shanghai 200234, China.


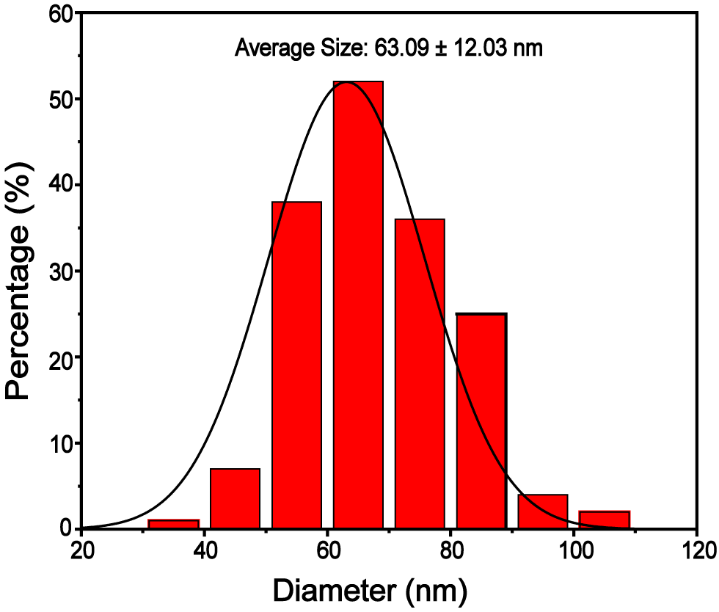


**Figure S1.** Size statistics of the Bi:Cu_2_O@HA NPs in a SEM image.


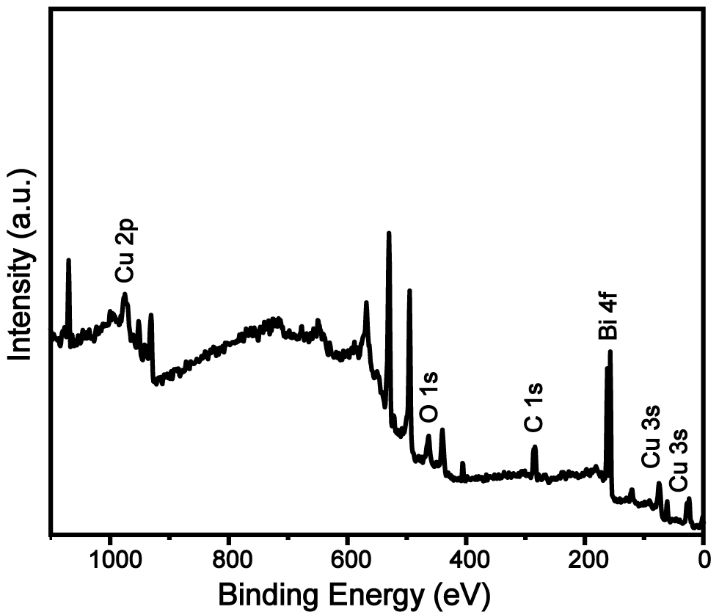


**Figure S2.** XPS spectrum of the Bi:Cu_2_O@HA NPs.


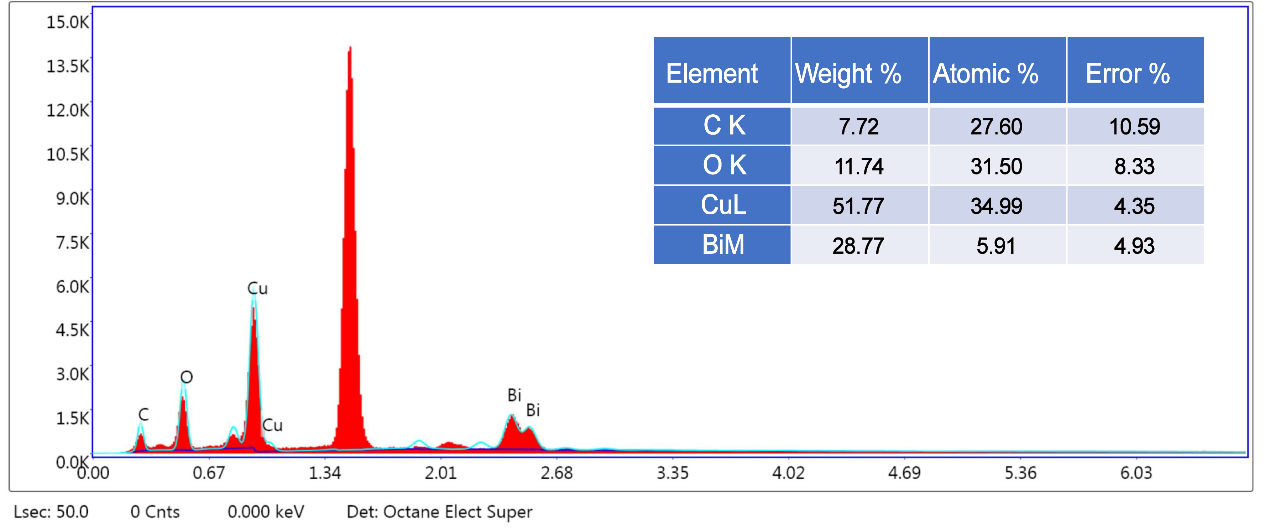


**Figure S3.** EDX analysis spectrum of the Bi:Cu_2_O@HA NPs.


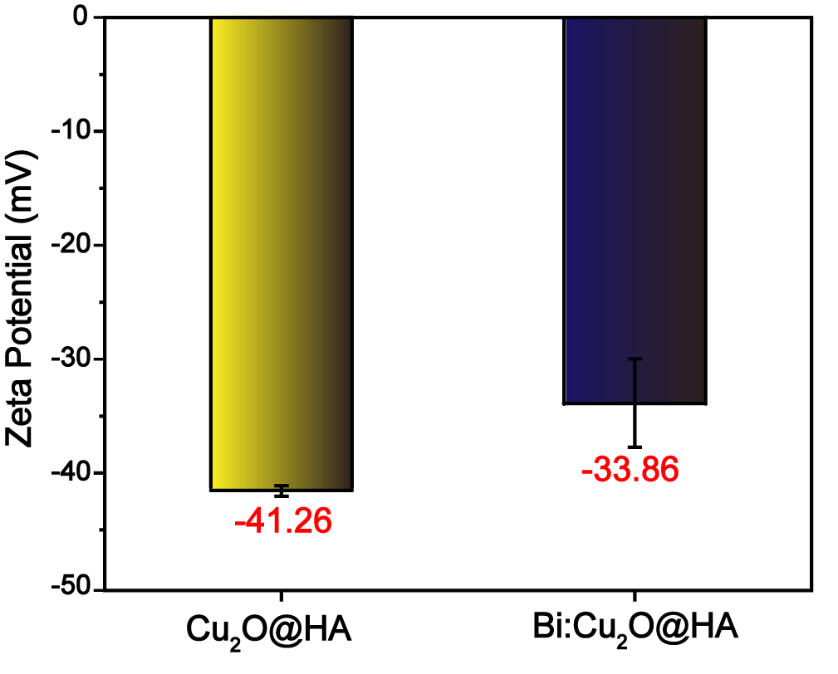


**Figure S4.** Zeta potentials of Cu_2_O@HA NPs and Bi:Cu_2_O@HA NPs.


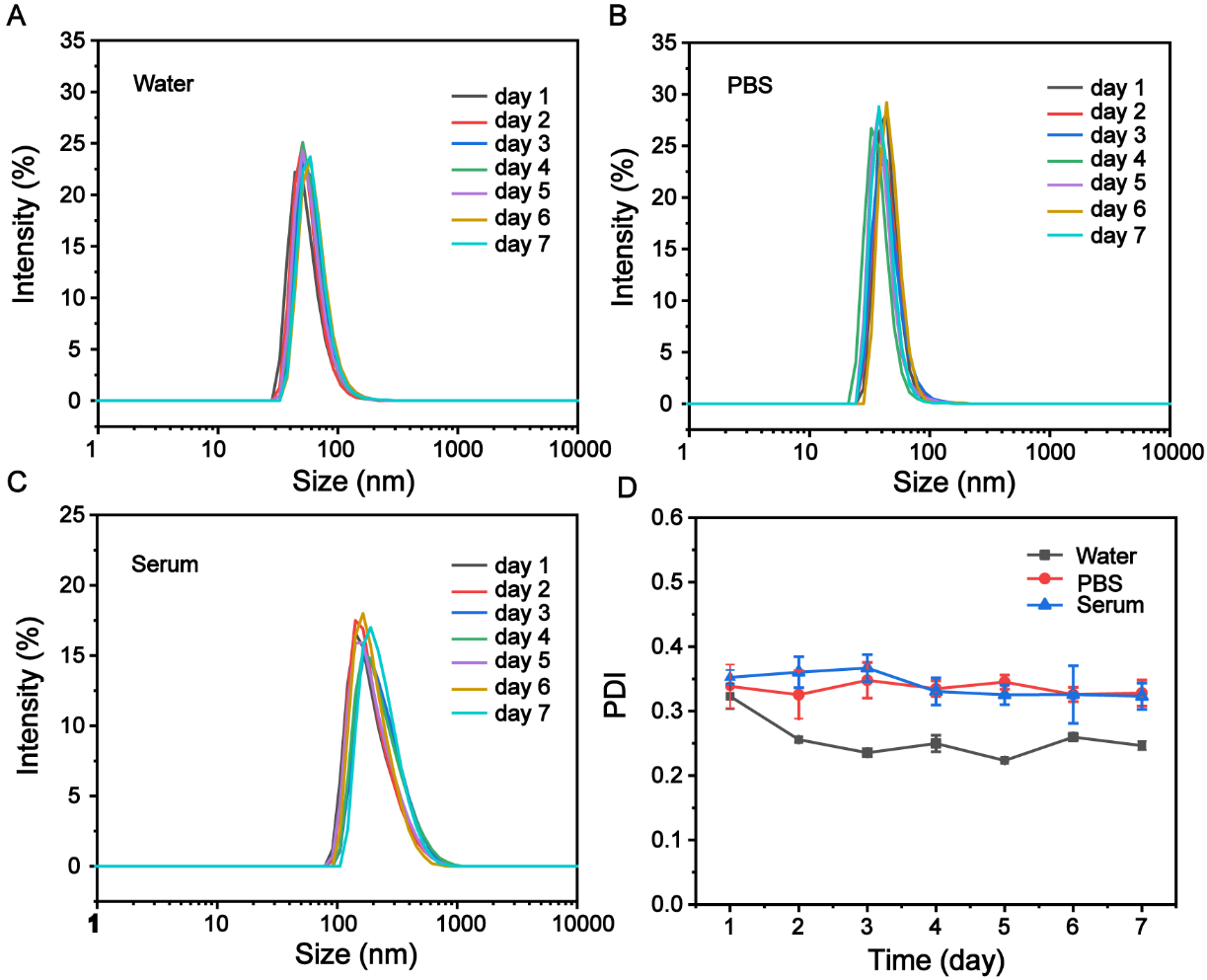


**Figure S5.** (A) Physiological stability of Bi:Cu_2_O@HA NPs in water. (B) Physiological stability of Bi:Cu_2_O@HA NPs in PBS. (C) Physiological stability of Bi:Cu_2_O@HA NPs in serum. (D) Corrsponding PDI change within 7 days.


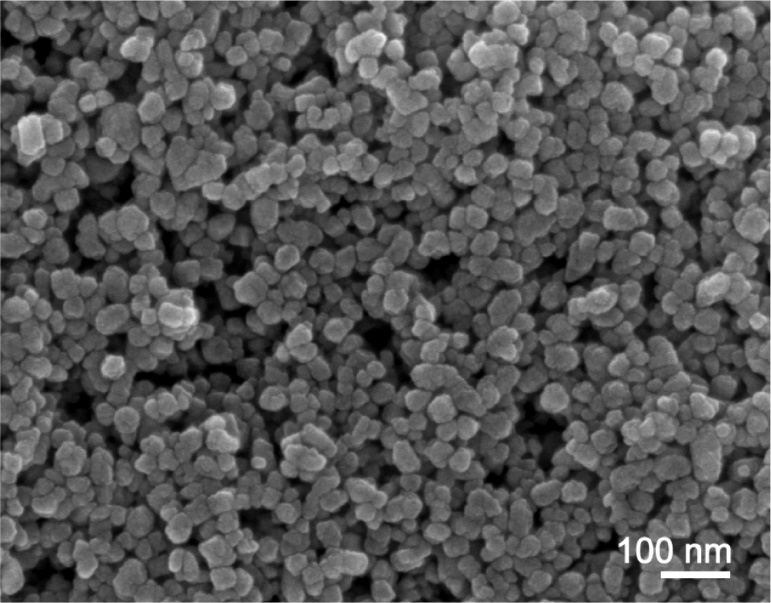


**Figure S6.** SEM of the Cu_2_O@HA NPs.


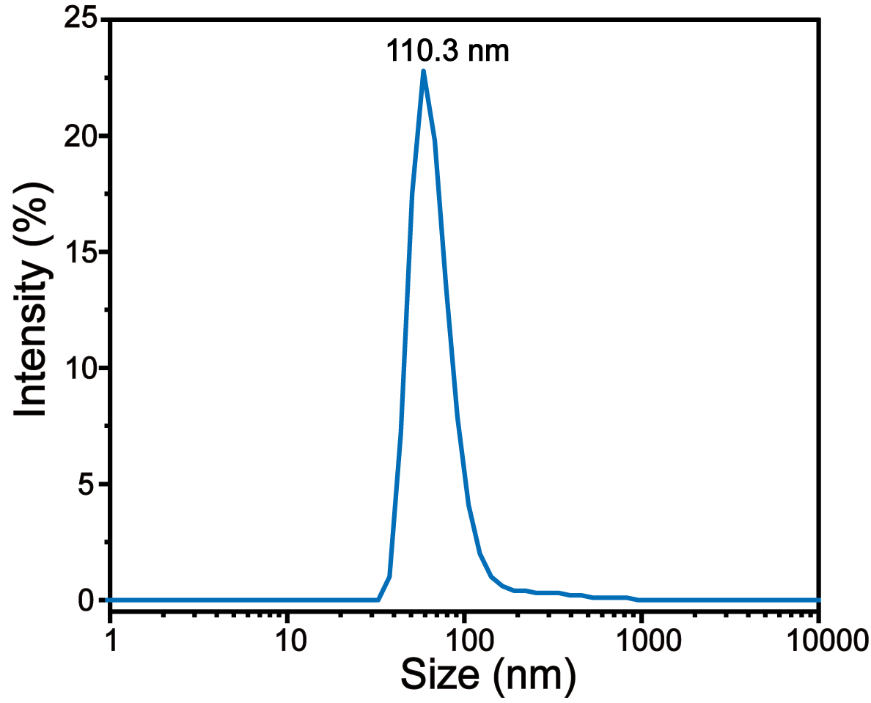


**Figure S7.** DLS size distributions of the Cu_2_O@HA NPs.


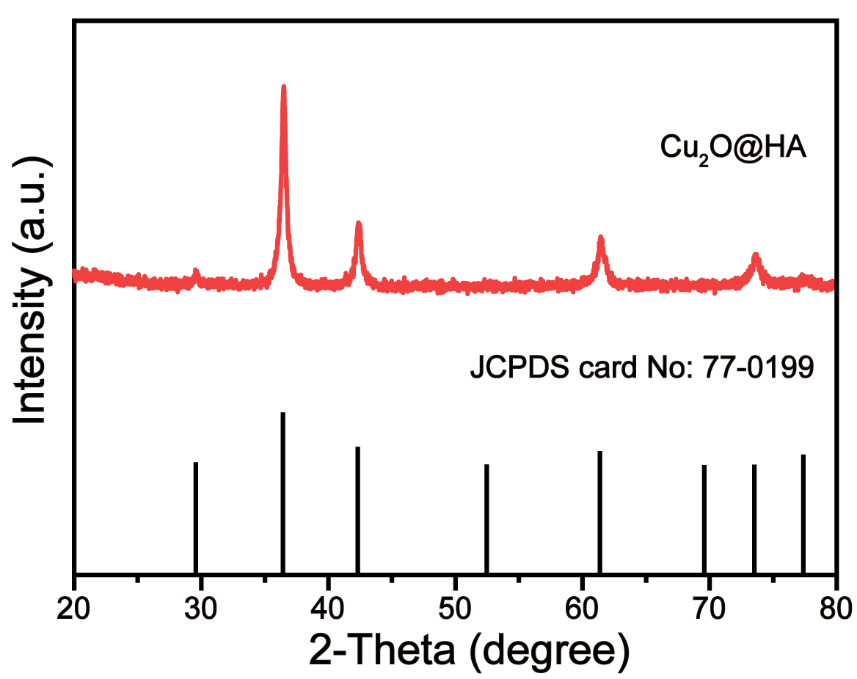


**Figure S8.** XRD pattern of Cu_2_O@HA NPs (red line).


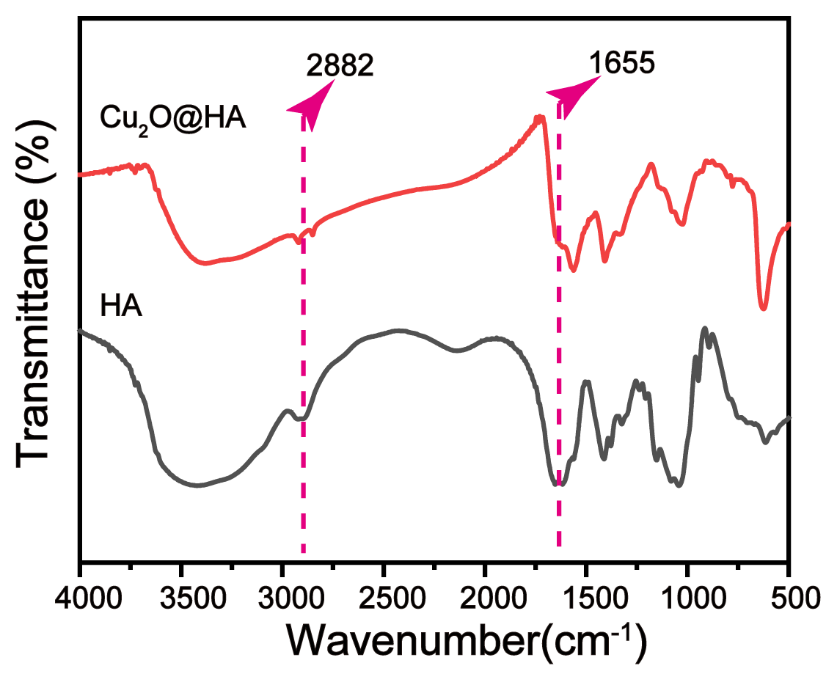


**Figure S9.** FT-IR spectra of HA (black line) and Cu_2_O@HA NPs (red line).


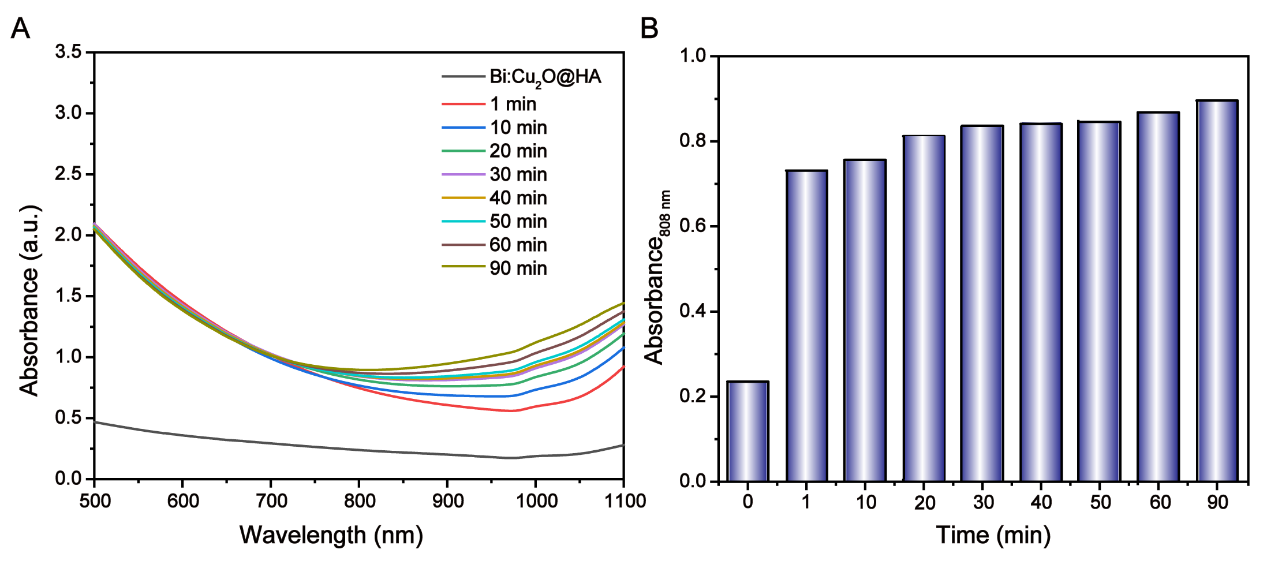


**Figure S10.** (A) UV-vis spectra of Bi:Cu_2_O@HA NPs reacted with NaHS for different time periods. (B) Corresponding absorption values at 808 nm.


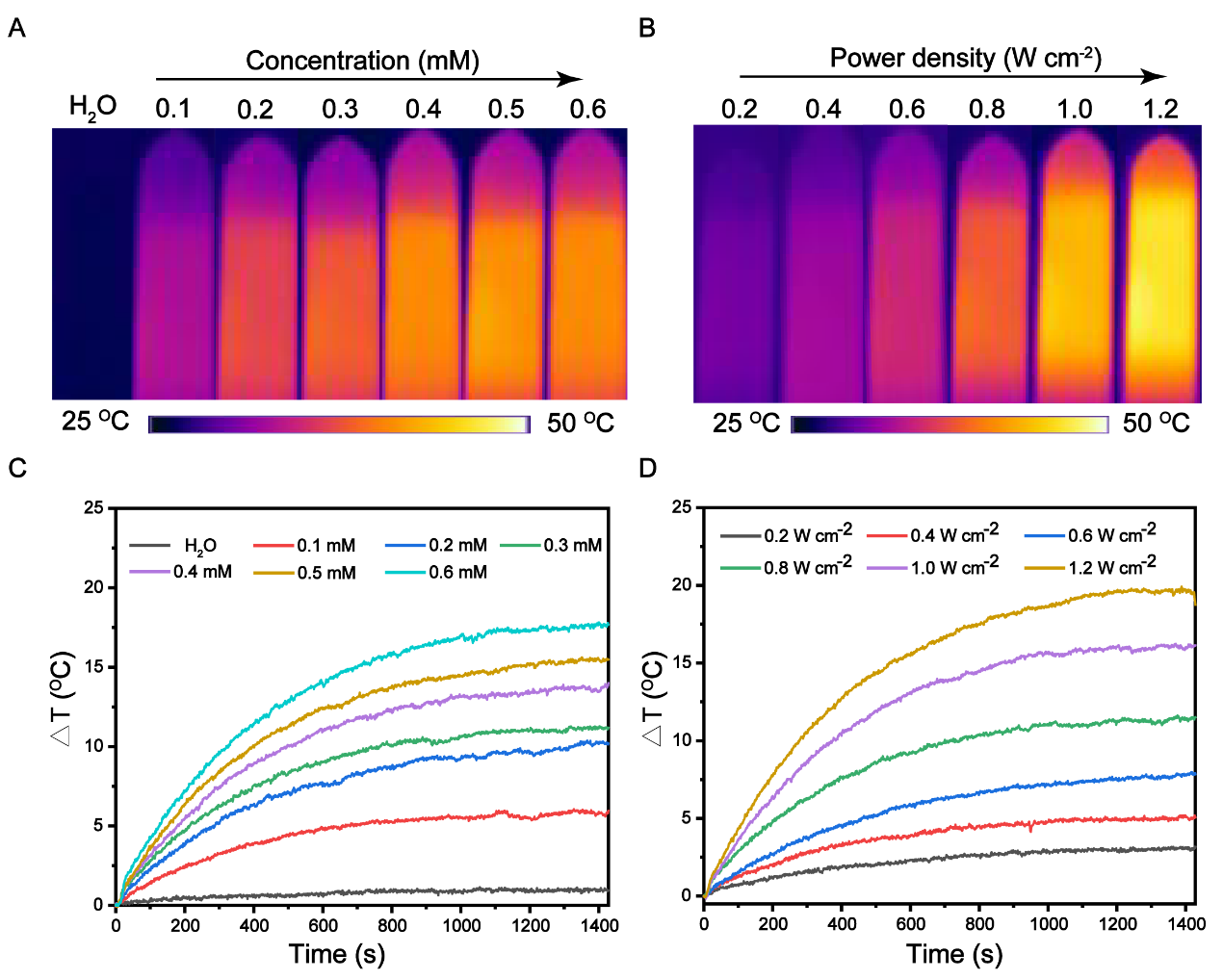


**Figure S11.** (A) Thermal images of the reaction of 4 mM NaHS with different concentrations of Bi:Cu_2_O@HA NPs (808 nm, 1 W/cm^2^). (B) Thermal images of the reaction of 4 mM NaHA with 0.5 mM Bi:Cu_2_O@HA NPs under different power density. (C) Plots of Δ*T* vs. time for different concentrations Bi:Cu_2_O@HA NPs reacted with 4 mM NaHS (808 nm, 1 W/cm^2^). (D) Plots of Δ*T* vs. time for 0.5 mM Bi:Cu_2_O@HA NPs reacted with 4 mM NaHS under different laser power densities.


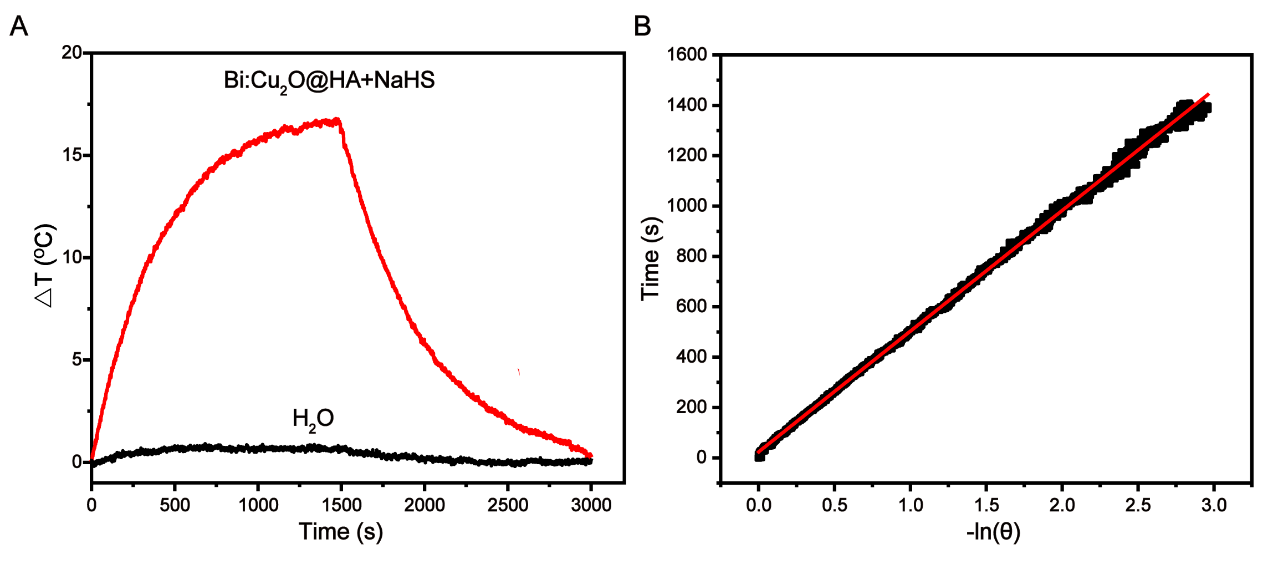


**Figure S12.** (A) Heating and cooling curves of water and Bi:Cu_2_O@HA+NaHS (4 mM) with the laser on and off. (B) corresponding time constant of the cooling curve. (The photothermal conversion efficiency of Bi:Cu_2_O@HA after reacted with NaHS was calculated to be 16.96%.)


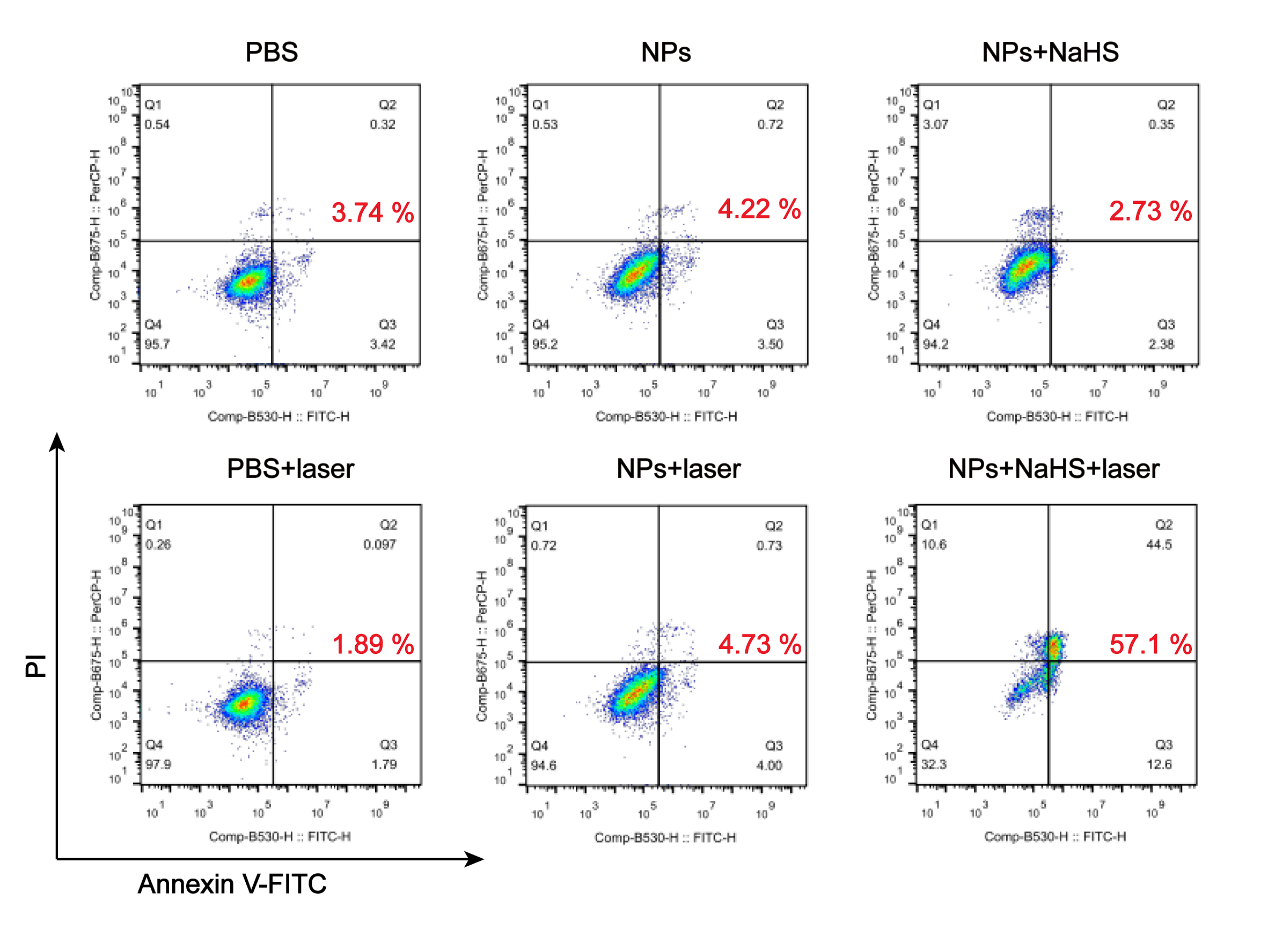


**Figure S13.** Apoptosis in CT26 cells after treatment in different groups.


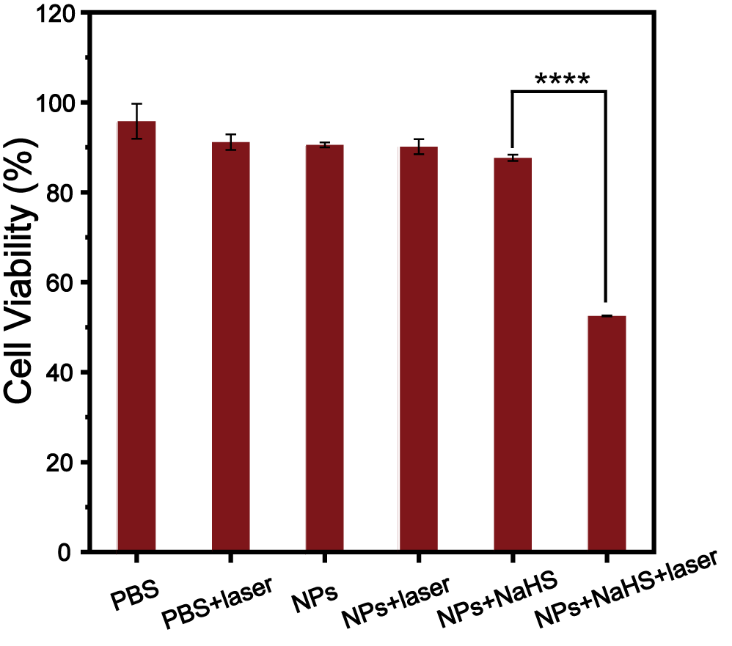


**Figure S14.** The cell viability of CT26 after treatment in different groups. Data are presented as means±SDs (n=5). ****p<0.0001.


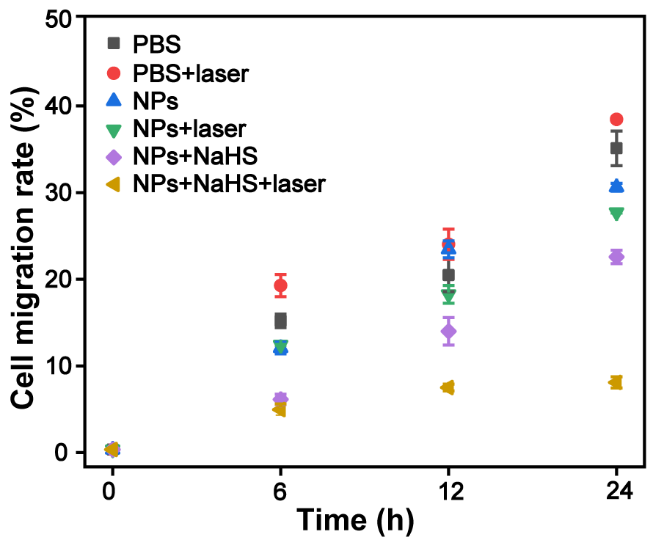


**Figure S15.** Cell migration rate of CT26 cells after treatment in different groups for 6 h, 12 h and 24 h.


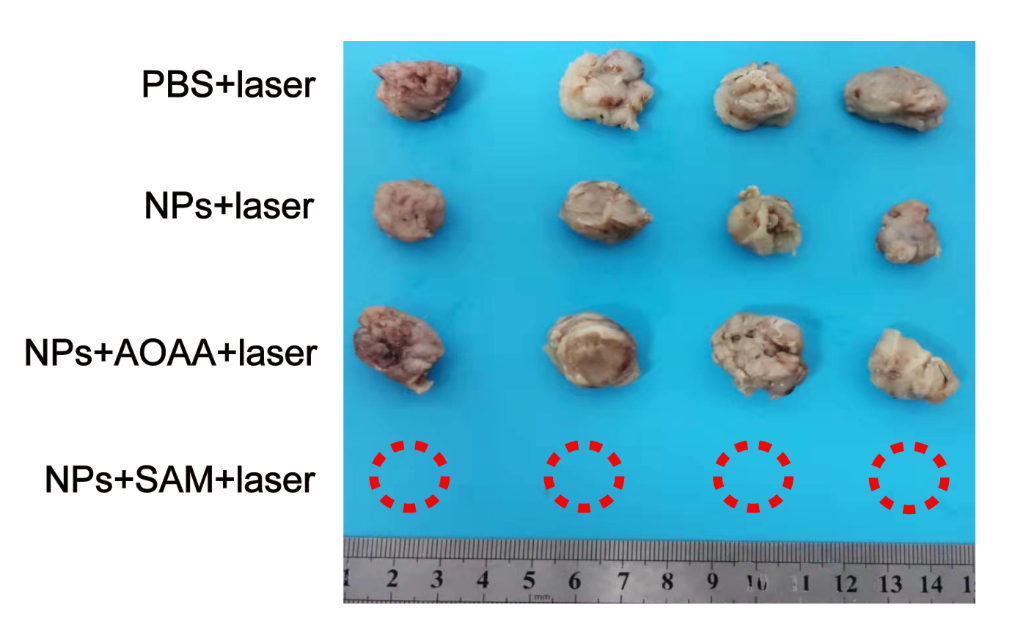


**Figure S16.** Photos of the tumors from mice in different groups after treatment.
